# TRIM35, a novel DNA-binding protein, epigenetically modifies H3 to promote HSPA6 transcription and suppress breast cancer progression

**DOI:** 10.1038/s41420-025-02770-9

**Published:** 2025-10-24

**Authors:** Xintao Jing, Fang Li, Jing Zhou, Jinyuan Zhang, Li Cao, Chen Guo, Qian Li, Hang Peng, Qiuyu Jiang, Xiaofei Wang, Yanke Chen, Jiangbo Ding, Dongdong Tong, Zhenghang Zhao, Chen Huang

**Affiliations:** 1 Department of Cell Biology and Genetics, School of Basic Medical Sciences, Xi’an Jiaotong University, Xi’an, Shaanxi China; 2 Institute of Basic Medical Sciences, Xi’an Medical University, Xi’an, Shaanxi China; 3 Northwest Women’s and Children’s Hospital, Xi’an, Shaanxi China; 4 Department of Gastroenterology, The First Affiliated Hospital of Xi’an Medical University, Xi’an, Shaanxi China; 5 Department of Otolaryngology Head and Neck Surgery, Tangdu Hospital, Fourth Military Medical University, Xi’an, Shaanxi China; 6 Biomedical Experimental Center, Xi’an Jiaotong University, Xi’an, Shaanxi China; 7 Department of Clinical Research, Xianyang Hospital of Yan’an University, Xianyang, China; 8 Department of Pharmacology, School of Basic Medical Sciences, Xi’an Jiaotong University Health Science Center, Xi’an, Shaanxi China

**Keywords:** Breast cancer, Post-translational modifications, Epigenetics

## Abstract

Tripartite Motif Containing 35 (TRIM35) is a well-characterized ubiquitin ligase with established roles in antiviral immunity, cancer metabolism, cardiovascular function, and tumor progression. However, its function as a DNA-binding protein has not been previously explored. In this study, we provide the first evidence that TRIM35 directly binds genomic promoters, thereby identifying it as a novel regulator of gene transcription. This finding opens new avenues for understanding the biological functions of TRIM35, expanding its potential role in cellular regulation. Furthermore, our results show that TRIM35 interacts with histone H3 (H3) and catalyzes its non-proteolytic ubiquitination, which serves as a recruitment signal for p300, leading to subsequent H3K27 acetylation and activation of gene transcription. Notably, among the genes regulated, Heat Shock Protein Family A (Hsp70) Member 6 (HSPA6) is significantly upregulated through TRIM35-mediated transcriptional regulation, which suppresses breast cancer progression and mediates TRIM35’s anti-tumor effect. Collectively, our findings reveal a previously unrecognized mechanism by which TRIM35 regulates gene expression through targeted epigenetic modification, providing new insights into its tumor-suppressive role in breast cancer.

## Introduction

Significant advances in diagnosis and treatment have been achieved through technological and scientific progress [1], yet breast cancer remains a leading cause of cancer-related mortality worldwide [2]. The development of targeted therapies has improved patient outcomes, but tumor heterogeneity and resistance to therapy persist as major challenges [3, 4]. Accumulating evidence underscores the critical role of epigenetic regulation in breast cancer progression and treatment [5, 6], particularly histone modifications [7, 8], which serve as key mechanisms for controlling gene expression.

Histone post-translational modifications (PTMs), including acetylation, methylation, and ubiquitination, dynamically regulate chromatin structure to modulate gene transcription [9, 10]. While acetylation and methylation involve the addition of chemical groups, ubiquitination involves the covalent attachment of ubiquitin proteins [11]. Although research on H3 ubiquitination is relatively limited compared to other modifications [12], its functional significance extends to protein degradation [13, 14], histone modification crosstalk [15], and DNA damage repair [16]. Given these diverse roles, further investigation into H3 ubiquitination mechanisms is essential. Acetylation, particularly p300/CBP-mediated H3K27ac, is widely associated with active transcription [17, 18], while crosstalk among different histone modifications adds complexity to transcriptional regulation [15, 19–23].

TRIM35 (Tripartite Motif Containing 35), a member of the TRIM family proteins, contains conserved RBCC domains: RING finger, B-boxes, and coiled-coil regions. The RING domain typically confers E3 ubiquitin ligase activity. Functionally, TRIM35 has been implicated in various biological processes, including cancer cell metabolism [24–26], cardiac remodeling [27], heart failure [28, 29], and antiviral immunity [30, 31]. In the context of cancer, its effects appear to be context-specific, as it promotes non-small cell lung cancer (NSCLC) progression [32] but inhibits diffuse large B-cell lymphoma (DLBCL) development [33]. Despite these established roles, TRIM35’s functions are primarily attributed to its ubiquitin ligase activity, leaving its potential involvement in DNA binding and epigenetic regulation largely uncharacterized.

This study introduces a novel function for TRIM35 as a DNA-binding protein that orchestrates epigenetic modifications to regulate gene expression in breast cancer cells. It preferentially targets gene promoters, interacts with histone H3, and catalyzes its non-proteolytic polyubiquitination. This modification serves as a key signal for p300 recruitment, leading to H3K27 acetylation and transcriptional activation. Consequently, TRIM35 promotes the expression of target genes, including HSPA6, further establishes its tumor-suppressive role in breast cancer. These findings provide new insights into TRIM35 as a critical epigenetic regulator, shedding light on a previously unexplored mechanism of gene expression control in cancer.

## Results

### TRIM35 is lowly expressed in breast cancer tissues and cells, and positively correlates with patient survival

**Fig. 1:**
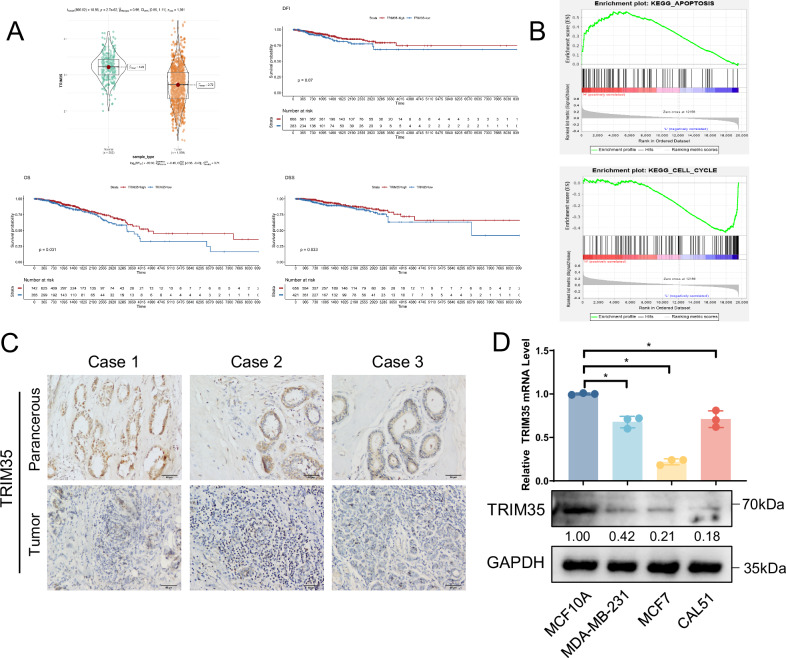
TRIM35 is lowly expressed in breast cancer tissues and cells, and positively correlates with patient survival. **A** TCGA and GETx databases were used to analyze the TRIM35 expression and correlation with patient survival. **B** Functional enrichment analysis of TRIM35 via GSEA. **C** Immunohistochemical identification of TRIM35 expression in breast cancer tissues and paracancerous tissues. **D** qRT-PCR and western blotting were performed to detect TRIM35 expression in breast cancer cells. Data are presented as mean ± SD (*n* = 3), * means *p* < 0.05.

Bioinformatics analysis using TCGA and GTEx datasets revealed that TRIM35 expression was significantly lower in breast cancer tissues (*n* = 1099) compared to normal breast tissues (*n* = 292) (Fig. 1A). Although TRIM35 expression showed no correlation with tumor stage or metastasis (Fig. S1A), higher TRIM35 levels were associated with prolonged overall survival (OS) and disease-specific survival (DSS) in patients (Fig. 1A). To explore the potential role of TRIM35 in breast cancer, we performed gene set enrichment analysis (GSEA), which indicated that TRIM35 expression was positively associated with apoptotic processes and negatively associated with the cell cycle (Fig. 1B). Immunohistochemistry results revealed that TRIM35 expression was downregulated in breast cancer tissues compared with that in corresponding paraneoplastic tissues (Fig. 1C). Western blotting and qRT-PCR analyses confirmed that TRIM35 expression was downregulated in breast cancer cell lines (MDA-MB-231, MCF7, and CAL51) compared to the normal breast epithelial cell line MCF10A (Fig. 1D).

### TRIM35 overexpression suppresses breast cancer cell proliferation in vitro and in vivo

**Fig. 2:**
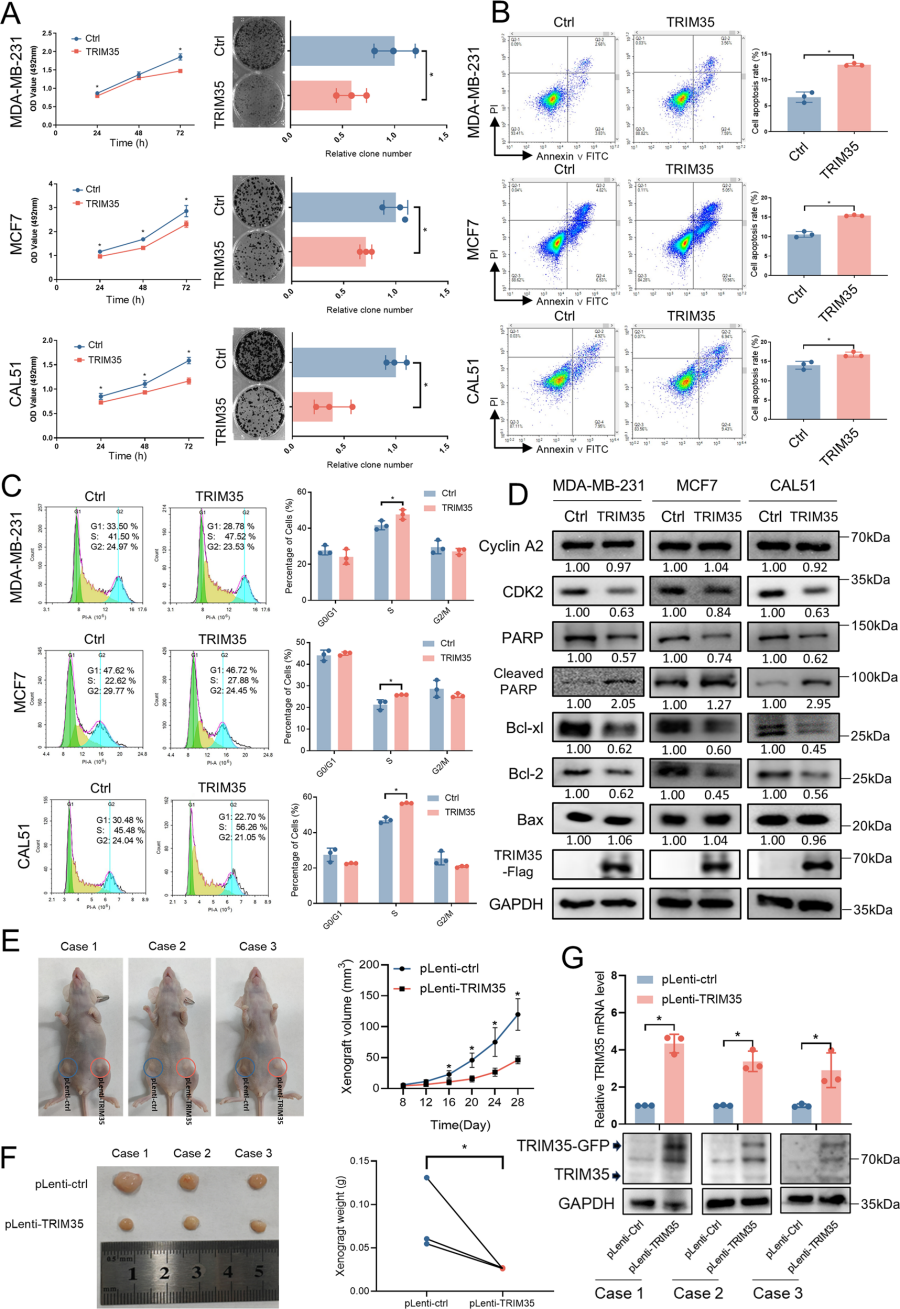
TRIM35 overexpression suppresses breast cancer cell proliferation in vitro and in vivo. **A** MTT and colony formation assays were employed to detect the impact of TRIM35 overexpression on the proliferation and the colony formation. Flow cytometry was used to detect the impact of TRIM35 overexpression on cell apoptosis (**B**) and cycle (**C**) of breast cancer cells. **D** Western blotting was utilized to detect the impact of TRIM35 overexpression on the protein level related to the cell apoptosis and cycle in breast cancer cells. **E** Imaging of tumor-bearing mice 28 days after injection (left), xenograft growth curves (right). **F** Imaging of xenografts 28 days after injection (left), xenograft weight statistics (right). **G** qRT-PCR and western blotting were used to detect the TRIM35 expression levels in xenografts. Data are presented as mean ± SD (*n* = 3), * means *p* < 0.05.

To further explore the biological effects of TRIM35 on breast cancer cells in vitro, we overexpressed TRIM35-Flag in MDA-MB-231, MCF7 and CAL51 cell lines (Fig. S1B). TRIM35 overexpression significantly inhibited cell proliferation and colony formation (Fig. 2A). However, wound-healing assays indicated that TRIM35 had no impact on the migratory ability of breast cancer cells (Fig. S1C). Flow cytometry analysis revealed that TRIM35 overexpression promoted apoptosis (Fig. 2B) and induced cell cycle arrest at the S phase (Fig. 2C). Western blotting further demonstrated a decrease in the apoptosis-suppressing proteins Bcl-xl and Bcl-2, an increase in PARP cleavage, and a reduction in the S-phase regulator CDK2 (Fig. 2D). These findings suggest that TRIM35 suppresses breast cancer cell proliferation by inducing cell cycle arrest and promoting apoptosis.

To further investigate the role of TRIM35 in breast cancer cell proliferation in vivo, we established the MCF7-pLenti-TRIM35 cell line, which stably overexpresses TRIM35 (Fig. S1D), along with the control MCF7-pLenti-ctrl cell line. These cells were injected subcutaneously into both inguinal flanks of nude mice, and tumor growth was monitored. Xenograft assays showed that tumor derived from MCF7-pLenti-TRIM35 cells grew more slowly than those from MCF7-pLenti-ctrl cells (Fig. 2E). Moreover, the average tumor volume and weight in the MCF7-pLenti-TRIM35 group were significantly lower than in the control group (Fig. 2F). RNA and protein analysis of the xenografts confirmed a significant upregulation of TRIM35 mRNA and protein (Fig. 2G) in the pLenti-TRIM35 group compared to controls.

These findings indicate that TRIM35 overexpression suppresses breast cancer cell growth both in vitro and in vivo.

### TRIM35 specifically binds to genomic promoters and interacts with H3

**Fig. 3:**
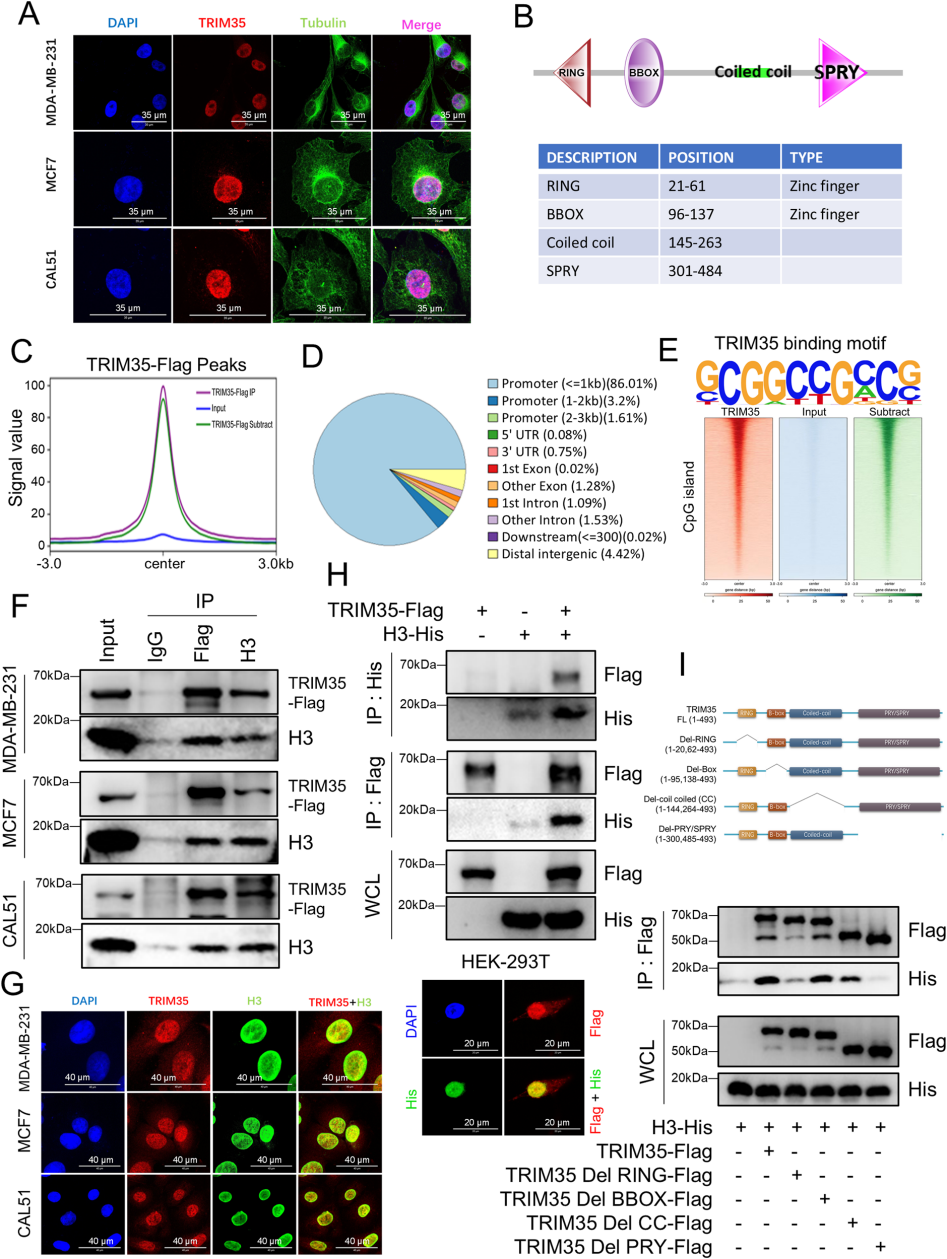
TRIM35 specifically binds to genomic promoters and interacts with H3. **A** Immunofluorescence staining was used to detect TRIM35 localization in breast cancer cells, scale bar = 35 μm. **B** The SMART website was used to visualize the TRIM35 protein structure. **C** Display of ChIP-seq signal values (The TRIM35-Flag subtract group is the result of subtracting the input group from the IP group). **D** The pie chart showing the distribution of TRIM35-Flag ChIP peaks across the human genome. **E** TRIM35 binding motif (up), and plotHeatmap showing TRIM35-Flag ChIP-seq signals on CpG islands (bottom). Co-IP and western blotting were performed to detect the TRIM35-H3 interactions (breast cancer cells (**F**), HEK-293T cells (**H**)). Immunofluorescence staining was utilized to detect the co-localization of TRIM35 with H3 (endogenous co-localization in breast cancer cells (**G**) (scale bar = 40 μm) and exogenous co-localization in HEK-293T cells (**H**) (scale bar = 20 μm)). **I** Schematic diagram of the TRIM35 truncation mutants (up). Co-IP and western blotting were used to identify key structural domains of TRIM35 interacting with H3 (bottom).

To investigate the molecular mechanisms by which TRIM35 inhibits breast cancer cell growth, we first examined its subcellular localization using immunofluorescence. The results showed that TRIM35 is predominantly localized in the nucleus (Fig. 3A). Ubiquitin ligase-mediated protein modification, which often leads to protein degradation, typically occurs in the cytoplasm. However, the robust nuclear expression of TRIM35 suggested that it may have additional functions beyond its well-characterized E3 ubiquitin ligase activity. By referring to the literature [31] and analyzing TRIM35’s domain architecture via the SMART website (https://smart.embl.de/), we identified four conserved domains: RING, BBOX, coiled-coil, and SPRY (Fig. 3B). Notably, the RING and BBOX domains are zinc finger structures, which are commonly associated with DNA binding. Based on this, we hypothesize that TRIM35 may function as a novel DNA-binding protein.

To further investigate the DNA-binding properties of TRIM35, we overexpressed TRIM35-Flag in MDA-MB-231 cells and performed ChIP assays using the Flag antibody, followed by high-throughput sequencing of the isolated chromatin. Peak annotation and analysis confirmed that the Flag-IP group was significantly enriched with chromatin compared to the input control (Fig. 3C). Notably, over 90% of the identified peaks were located within promoter regions of the human genome (Fig. 3D). Motif analysis using HOMER [34] (http://homer.ucsd.edu/homer/) revealed that TRIM35 preferentially binds to DNA sequences with high GC content (Fig. 3E). Additionally, the TRIM35-enriched peaks highly overlapped with CpG islands (Fig. 3E), with 77% of these peaks localized within CpG islands in gene promoter regions (data not shown). These results suggest that TRIM35 may play a role in gene transcription regulation. To determine whether this regulatory function is mediated through direct transcription factor activity, histone modification, or alternative pathways, we conducted further investigations.

Given TRIM35’s role as a ubiquitin ligase, we focused on histone modifications as a potential mechanism for regulating gene expression. Previous studies have shown that TRIM35 can monoubiquitinate lysine 120 (K120) on histone H2B in postnatal mature cardiomyocytes, thereby promoting chromatin remodeling [28]. As histone H3 is a principal scaffold for multiple transcription-associated modifications [35] and has well-documented cross-talk with ubiquitination [36, 37], we further examined whether TRIM35 also interacts with histone H3. Co-IP experiments demonstrated that TRIM35 co-precipitates with histone H3 in three different breast cancer cell lines (Fig. 3F). Additionally, immunofluorescence assays revealed that TRIM35 co-localizes with histone H3 in these cells (Fig. 3G). To validate these findings, we transfected TRIM35-Flag and H3-His into HEK-293T cells and performed both immunoprecipitation and immunofluorescence assays using tag-specific antibodies. The results were consistent with those obtained from the breast cancer cell lines (Fig. 3H). To further investigate the structural domains of TRIM35 that mediate its interaction with H3, we constructed TRIM35 deletion mutants (Del-RING, Del-BBOX, Del-coiled coil, and Del-SPRY) (Fig. 3I). Co-transfection of these mutants with H3-His into HEK-293T cells, followed by Co-IP analysis revealed that deletion of the RING and SPRY domains significantly weakened TRIM35’s interaction with H3 (Fig. 3I).

### TRIM35 polyubiquitinates H3, and the ubiquitination signal recruits p300 to acetylate H3K27

**Fig. 4:**
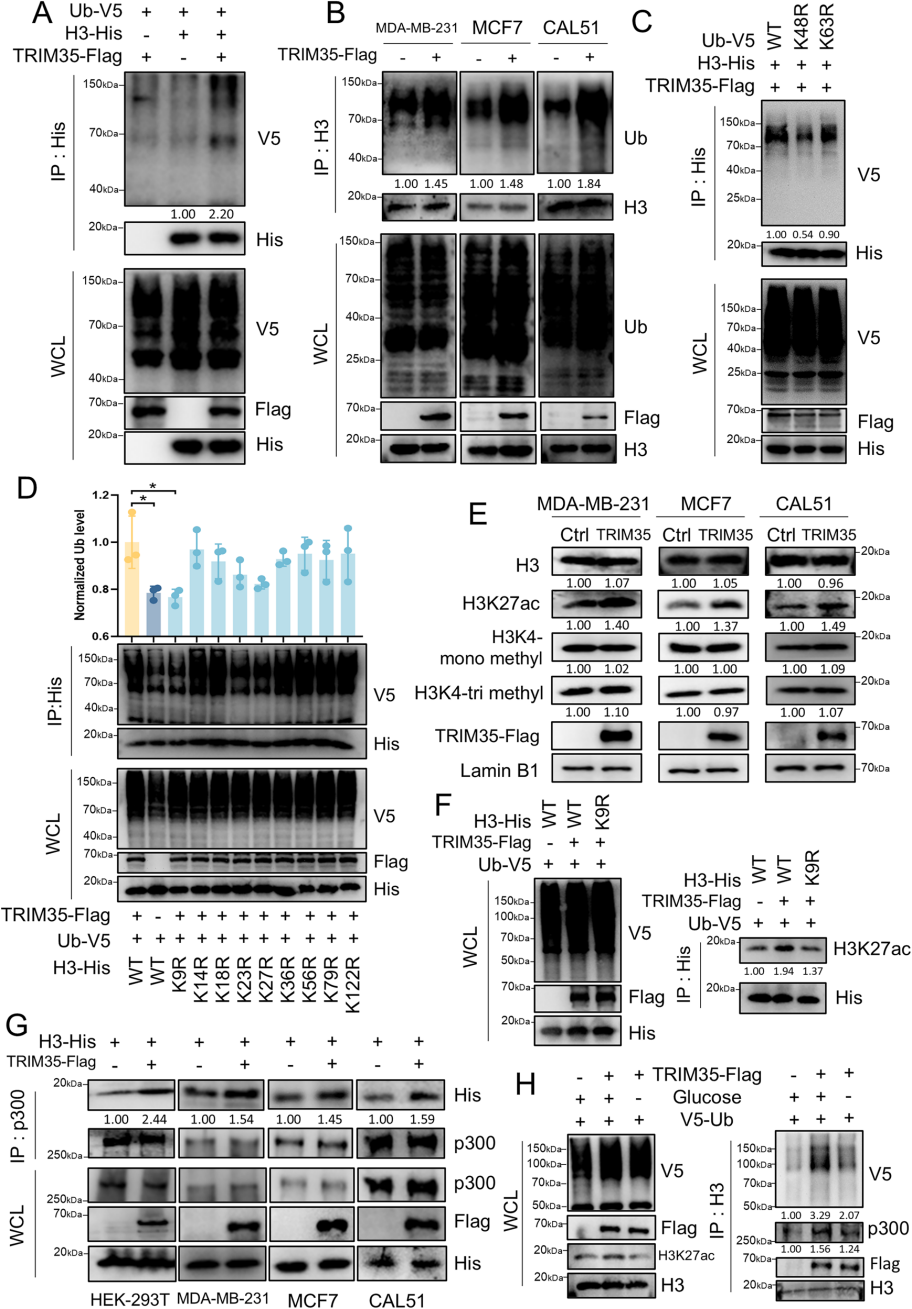
TRIM35 polyubiquitinates H3, and the ubiquitination signal recruits p300 to acetylate H3K27. Co-IP and western blotting were performed to detect H3 polyubiquitination mediated by TRIM35 in breast cancer cells (**B**) and HEK-293T cells (**A**). **C** Co-IP and western blotting were used to detect the polyubiquitination form of H3 mediated by TRIM35 in HEK-293T cells. **D** Co-IP and western blotting were utilized to identify the H3 ubiquitination sites mediated by TRIM35 in HEK-293T cells, with ubiquitination levels normalized to His in the IP group. Data are presented as mean ± SD (*n* = 3), * means *p* < 0.05. **E** Western blotting was used to detect the effect of TRIM35 overexpression on H3 protein levels and several epigenetically modified H3 proteins. **F** Co-IP and western blotting were performed to confirm TRIM35 overexpression-induced H3K27 acetylation results from H3 ubiquitination in HEK-293T cells. **G** Co-IP and western blotting were used to confirm TRIM35 overexpression enhanced the p300-H3 interaction. **H** Co-IP and western blotting were utilized to confirm the enhanced p300-H3 interaction induced by TRIM35 overexpression is a result of H3 ubiquitination. At 32 h post-transfection, HEK-293T cells in the third group were subjected to incubation in glucose-free medium for 4 h prior to protein extraction and subsequent analysis.

To further investigate the epigenetic modification of histone H3 mediated by TRIM35, we performed Co-IP and western blotting experiments, which revealed that H3 accumulates more ubiquitin following TRIM35 overexpression (Fig. 4A, B). Previous studies have shown that H3 can undergo mono-, di-, tri or polyubiquitination [15, 38]. Our results indicated that TRIM35 specifically mediates polyubiquitination of H3. Polyubiquitination typically forms chains linked through lysine residues at either Ub-K48 or Ub-K63. To identify the specific type of polyubiquitination catalyzed by TRIM35, we used mutant ubiquitin expression vectors with lysine 48 (K48) and lysine 63 (K63) mutated to arginine (Ub-K48R-V5 and Ub-K63R-V5). Compared to wild-type ubiquitin (Ub-WT-V5), we observed a significant reduction in H3 polyubiquitination when K48 was mutated, whereas mutation of K63 had no significant effect on H3 ubiquitination (Fig. 4C). Next, we aimed to identify the specific ubiquitination sites on H3 that are dependent on TRIM35. Previous studies have identified potential H3 ubiquitination sites through large-scale quantitative proteomics (Fig. S2A). We constructed several H3 expression vectors, each with a single-point mutation at the predicted ubiquitination sites. Co-IP and western blotting experiments revealed that TRIM35-mediated polyubiquitination of H3 was significantly reduced when H3K9 was mutated (Fig. 4D).

Compared with acetylation and methylation, research on histone H3 ubiquitination is relatively limited, yet it spans a wide range of functional aspects. Existing reports focusing on its roles in promoting protein degradation, mediating histone epitope modifications, and regulating DNA replication and damage repair [12, 39]. To explore this further, we assessed the protein levels of H3 and found that TRIM35 overexpression did not alter H3 protein levels (Fig. 4E). We then tested several lab-available antibodies targeting histone modifications and found that TRIM35 overexpression increased the acetylation levels of H3K27, while it had no effect on H3K4 mono- or trimethylation (Fig. 4E). Additionally, the increase in H3K27 acetylation induced by TRIM35 overexpression was partially reversed when H3K9 was mutated, pointing to the role of TRIM35-mediated H3 ubiquitination in the upregulation of H3K27ac (Fig. 4F).

p300 is a well-known histone acetyltransferase. Although Co-IP experiments showed that TRIM35 and p300 could not be mutually enriched (Fig. S2B), overexpression of TRIM35 facilitated increased binding of p300 to H3 (Fig. 4G), which in turn led to enhanced acetylation of H3K27. Moreover, when H3 ubiquitination was inhibited by glucose removal [15], the binding of p300 to H3 was weakened (Fig. 4H).

In summary, TRIM35 mediates K48-polyubiquitination at H3K9, and this ubiquitination signal subsequently recruits p300, which acetylates H3K27.

### Integrative analysis of ChIP-seq and RNA-seq identifies TRIM35-regulated targets

**Fig. 5:**
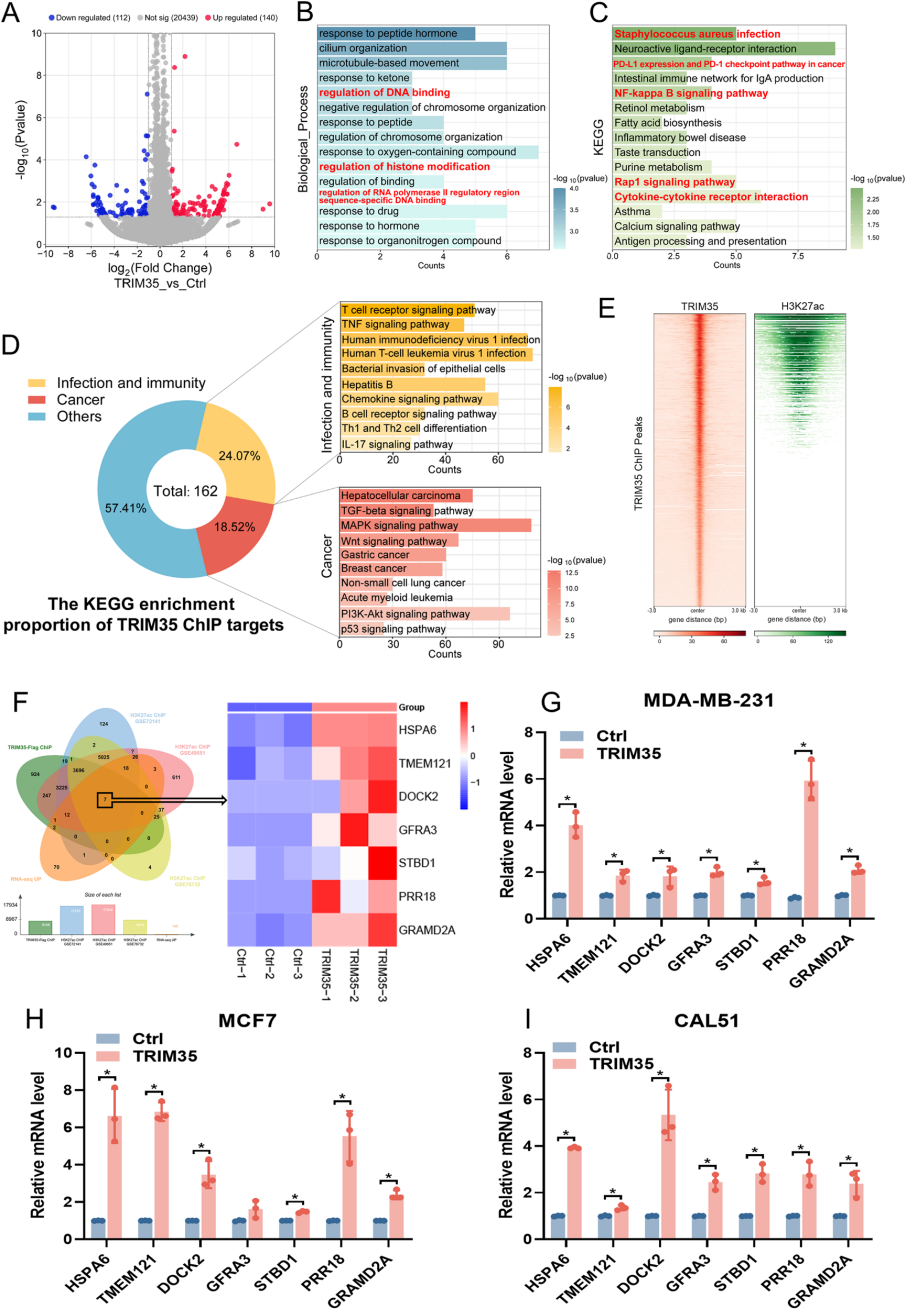
Integrative analysis of ChIP-seq and RNA-seq identifies TRIM35-regulated targets. **A** Volcano plot depicting DEGs in MDA-MB-231 cells with TRIM35 overexpression versus control. GO (**B**) and KEGG (**C**) enrichment analysis of DEGs following TRIM35 overexpression, with the top 15 terms based on *p* value. **D** KEGG enrichment analysis and statistics of TRIM35-Flag ChIP targets, highlighting cancer-related terms and immunity/infection pathways. **E** The plotHeatmap illustrating the TRIM35-Flag ChIP-seq signals at the H3K27ac position. **F** Venn diagrams showing overlapping targets from RNA-seq UP, TRIM35-Flag ChIP-seq, and H3K27ac ChIP-seq (GSE72141, GSE49651, GSE76732) (left), with a heatmap of RNA expression in overlapping targets following TRIM35 overexpression (right). qRT-PCR validation of overlapping target expression post-TRIM35 overexpression in MDA-MB-231 (**G**), MCF7 (**H**), and CAL51 (**I**) cells. Data are presented as mean ± SD (*n* = 3), * means *p* < 0.05.

H3K27ac is widely recognized as a marker of active transcription [40]. To investigate the transcriptional changes induced by TRIM35 overexpression, we performed RNA-seq on control and TRIM35-overexpressing cells. Our analysis identified 112 downregulated genes and 140 upregulated genes (Fig. 5A). Functional enrichment analysis of these differentially expressed genes (DEGs) revealed associations with DNA binding, histone modification, and RNA polymerase binding to DNA (Fig. 5B), consistent with our earlier findings. Furthermore, KEGG pathway enrichment analysis indicated that the DEGs were related to microbial infection, tumor immunity, and inflammatory responses (Fig. 5C). KEGG analysis of TRIM35 targets also revealed associations with various tumors and immune responses to infection (Fig. 5D). To further explore the relationship between TRIM35 and H3K27ac, we compared our TRIM35 ChIP-seq data with publicly available H3K27ac ChIP-seq data (61914) from the Cistrome Data Browser (Cistrome DB). Our comparison showed partial overlap between the TRIM35 binding peaks and those of H3K27ac (Fig. 5E).

To identify the targets regulated by TRIM35 as described above, we analyzed overlapping genes from the H3K27ac ChIP-seq data, TRIM35 ChIP-seq data, and the upregulated genes identified in the RNA-seq data (Fig. 5F). Seven genes were identified (Fig. 5F), which we hypothesized to be transcriptionally regulated through the mechanism outlined earlier—that is, TRIM35 binds to the promoters of these genes, modifies H3 epigenetically, and thereby enhances gene transcription. Furthermore, these seven genes were significantly upregulated following TRIM35 overexpression (Fig. 5G–I). Based on the significance of the differences and the expression levels, we selected HSPA6 for further validation.

### TRIM35 binds to the HSPA6 promoter and epigenetically modifies H3 to promote HSPA6 transcription

**Fig. 6:**
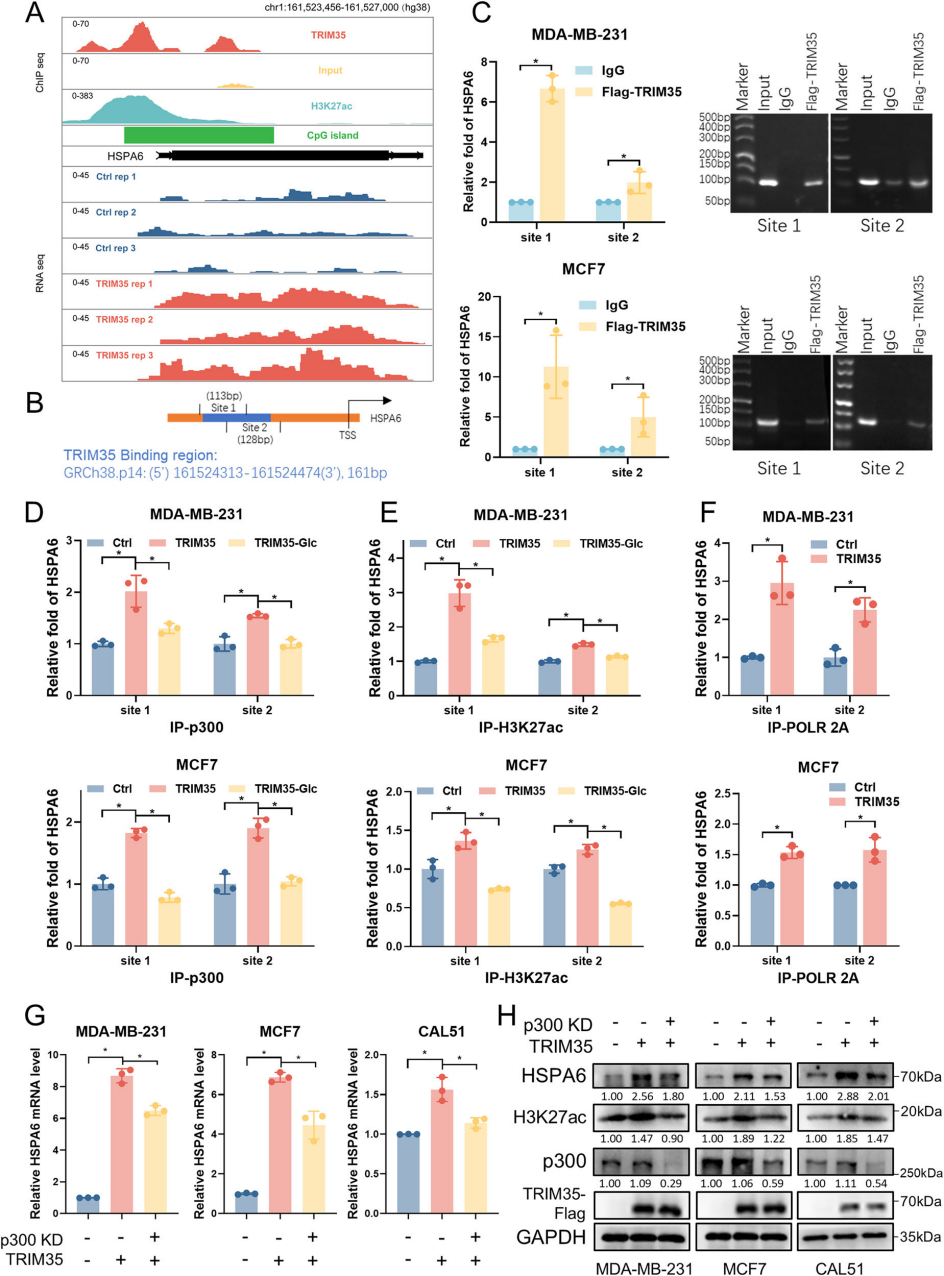
TRIM35 binds to the HSPA6 promoter and epigenetically modifies H3 to promote HSPA6 transcription. **A** IGV tracks showing TRIM35-Flag ChIP-seq and RNA-seq signals (under TRIM35 overexpression) on the HSPA6 promoter in MDA-MB-231 cells. **B** Schematic diagram illustrating the TRIM35 binding region on the HSPA6 promoter. **C** ChIP-qPCR and agarose gel electrophoresis were used to verify the binding of TRIM35 to the HSPA6 promoter. ChIP-qPCR analysis of p300 binding (**D**) and H3K27ac modification (**E**) at the HSPA6 promoter upon TRIM35 overexpression and glucose depletion. **F** ChIP-qPCR was conducted to detect the effect of TRIM35 overexpression on POLR 2A binding at the HSPA6 promoter. qRT-PCR (**G**) and western blotting (**H**) were used to detect the rescue effect of p300 knockdown on HSPA6 upregulation induced by TRIM35 overexpression. Data are presented as mean ± SD (*n* = 3), * means *p* < 0.05.

We first visualized the ChIP-seq results and observed that TRIM35 binding peaks were present in the HSPA6 promoter, overlapping with H3K27ac modification (Fig. 6A). Based on the TRIM35 ChIP-seq data, we pinpointed the specific region where TRIM35 binds to the HSPA6 promoter (Fig. 6B), dividing this region into two segments to design primers for subsequent validation. ChIP-qPCR results confirmed that TRIM35 indeed binds to the HSPA6 promoter (Fig. 6C). Further analysis showed that, following TRIM35 overexpression, the HSPA6 promoter exhibited increased p300 binding (Fig. 6D) and enhanced H3K27ac modification (Fig. 6E), accompanied by greater RNA polymerase II (POLR2A) binding (Fig. 6F). However, upon glucose removal, which suppresses H3 ubiquitination, the increased p300 binding and H3K27ac modification following TRIM35 overexpression at the HSPA6 promoter were attenuated (Fig. 6D, E). Although HSPA6 expression was strongly induced by the nutrient stress [41] caused by glucose deprivation (Fig. S3A), these epigenetic changes still occurred. This demonstrated that the increased p300 binding and H3K27ac levels at the HSPA6 promoter following TRIM35 overexpression were indeed caused by TRIM35-mediated H3 ubiquitination. Moreover, knockdown of p300 (Fig. S3B) partially rescued the transcriptional activation of HSPA6 caused by TRIM35 overexpression (Fig. 6G), with consistent changes observed in protein levels (Fig. 6H).

Furthermore, we examined the expression of HSPA6 and related regulatory molecules in tumor tissues from the previous TRIM35-overexpression xenograft models. The results showed that both HSPA6 mRNA (Fig. S3C) and protein levels (Fig. S3D) were elevated in the TRIM35-overexpression group, with increased H3K27ac protein levels but no significant change in p300 protein levels (Fig. S3D). These findings are consistent with our in vitro results, supporting the role of TRIM35 in promoting H3K27 acetylation and transcriptional activation of HSPA6 in vivo.

In summary, TRIM35 binds to the HSPA6 promoter and mediates H3 ubiquitination. This ubiquitination signal recruits p300, which then acetylates H3K27, ultimately promoting HSPA6 transcription.

### HSPA6 suppresses breast cancer cell growth and mediates TRIM35’s anti-tumor effect

**Fig. 7:**
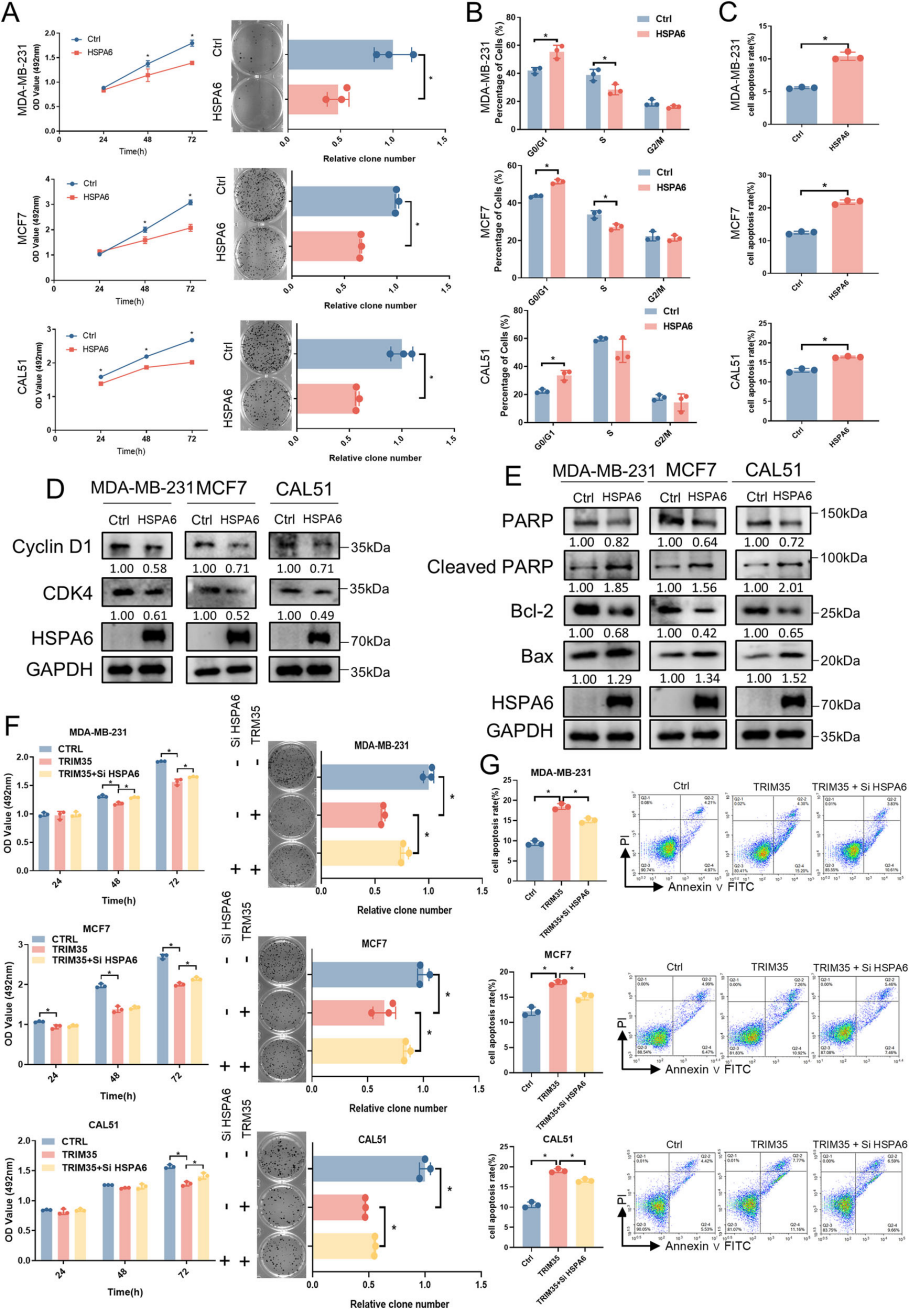
HSPA6 suppresses breast cancer cell growth and mediates TRIM35’s anti-tumor effect. **A** MTT and colony formation assays were employed to assess the impact of HSPA6 overexpression on breast cancer cell proliferation and colony formation. Statistical analysis of the effects of HSPA6 overexpression on the cell cycle (**B**) and apoptosis (**C**) of breast cancer cells. Western blotting analysis of the protein levels associated with the cell cycle (**D**) and apoptosis (**E**) in response to HSPA6 overexpression. **F** MTT and colony formation assays were used to verify the ability of HSPA6 knockdown to partially reverse the inhibitory effect of TRIM35 overexpression on breast cancer cell proliferation. **G** Flow cytometry analysis of cell apoptosis confirming that HSPA6 knockdown partially reverses the pro-apoptotic effects of TRIM35 overexpression. Data are presented as mean ± SD (*n* = 3), * means *p* < 0.05.

Subsequently, we investigated the role of HSPA6 in breast cancer cells. Kaplan-Meier survival analysis from the Gene Expression Profiling Interactive Analysis 2 (GEPIA2) database showed that high expression of HSPA6 was positively associated with longer disease-free survival (DFS) in breast cancer patients (Fig. S4A). MTT and colony formation assays revealed that overexpression of HSPA6 (Fig. S4B) inhibited both cell proliferation and colony forming in breast cancer cells (Fig. 7A). Flow cytometry analysis of the cell cycle and apoptosis showed that HSPA6 overexpression arrested the cell cycle at the G1 phase (Figs. 7B and S4C) and promoted apoptosis in breast cancer cells (Figs. 7C and S4D). Additionally, the levels of G1 phase proteins CDK4 and Cyclin D1 were reduced (Fig. 7D). The anti-apoptotic protein Bcl-2 was also downregulated, while the pro-apoptotic protein Bax was upregulated, accompanied by increased PARP cleavage (Fig. 7E). However, HSPA6 overexpression had no effect on breast cancer cell migration (Fig. S4E). These findings suggest that the functional role of HSPA6 in breast cancer cells aligns with that of TRIM35.

Next, we explored whether silencing HSPA6 could reverse the inhibitory effects of TRIM35 overexpression on breast cancer cells. MTT and colony formation assays revealed that silencing HSPA6 (Fig. S4F, G) partially rescued the inhibitory effect of TRIM35 overexpression on breast cancer cell proliferation (Fig. 7F). Flow cytometry analysis of apoptosis showed that silencing HSPA6 partially reversed the pro-apoptotic effect of TRIM35 overexpression in breast cancer cells (Fig. 7G). Notably, we also observed that HSPA6 overexpression synergistically enhanced TRIM35-mediated tumor suppression (Fig. S4H).

In summary, HSPA6 suppresses breast cancer cell growth and mediates TRIM35’s anti-tumor effect.

## Discussion

Our functional experiments establish that TRIM35 acts as a tumor suppressor in breast cancer progression, corroborating prior reports of its anti-tumor activity [26, 42]. Furthermore, our study provides novel insights into the function of TRIM35 as an epigenetic regulator in breast cancer. While previous studies have primarily focused on its role as a ubiquitin ligase in viral infections and other cellular processes [26, 29, 30], our findings reveal that TRIM35 can also bind to genomic promoters and regulate transcription through histone modifications.

Our findings demonstrate that TRIM35 interacts with histone H3 via its RING (ubiquitin ligase catalytic site [31, 43]) and SPRY domains (protein-protein interaction domain [44]). Further investigation revealed that TRIM35 mediates H3 K48-polyubiquitination, a modification that typically marks proteins for proteasomal degradation [45]. However, in present study, we observed that TRIM35-induced K48 polyubiquitination does not lead to histone degradation but instead serves as a regulatory signal. This is similar to previous studies showing that K48-linked polyubiquitination can have non-proteolytic functions, such as stabilizing other ubiquitin chains [46] or modulating transcriptional activity [47]. Our findings provide new evidence of nonproteolytic functions of K48-linked polyubiquitination [48].

By integrating ChIP-seq and RNA-seq data, we identified HSPA6 as a key downstream target of TRIM35. ChIP-qPCR experiments confirmed that TRIM35 binds directly to the HSPA6 promoter and induces H3 epigenetic modifications, leading to transcriptional activation. Furthermore, consistent with prior studies demonstrating that HSPA6 silencing promotes BT-549 cell proliferation [49], our functional assays confirm that HSPA6 overexpression inhibits breast cancer cell growth. Importantly, our findings suggest that TRIM35-mediated regulation of HSPA6 represents a novel mechanism through which TRIM35 can influence cancer progression.

Our functional enrichment analysis revealed that TRIM35 is linked not only to cancer but also to tumor immunity and inflammation. This suggests that TRIM35’s role in regulating gene expression may extend beyond direct effects on tumor cells to include modulation of the tumor immune microenvironment. Further studies are needed to explore how TRIM35 influences anti-tumor immunity and whether this could be exploited for therapeutic purposes.

The present study raises several intriguing questions about TRIM35’s role in epigenetic regulation. For example, the enrichment of TRIM35 ChIP peaks on CpG islands suggests a potential relationship with DNA methylation that warrants further investigation. Additionally, our study has focused on the effects of TRIM35-mediated H3 ubiquitination on H3K27ac. Nevertheless, this modification may also impact other histone modifications or chromatin states, which warrants further investigation in future studies. Furthermore, we focused on triple-negative (MDA-MB-231, CAL51) and luminal-type (MCF-7) breast cancer cell lines, which together represent the majority of breast cancer cases (>80%) and cover key phenotypic features of these subtypes [50, 51]. Building on the above findings, future investigations will be extended to include HER2-positive models, aiming to provide a more comprehensive understanding of TRIM35’s role across the full spectrum of breast cancer subtypes.

## Conclusions

**Fig. 8:**
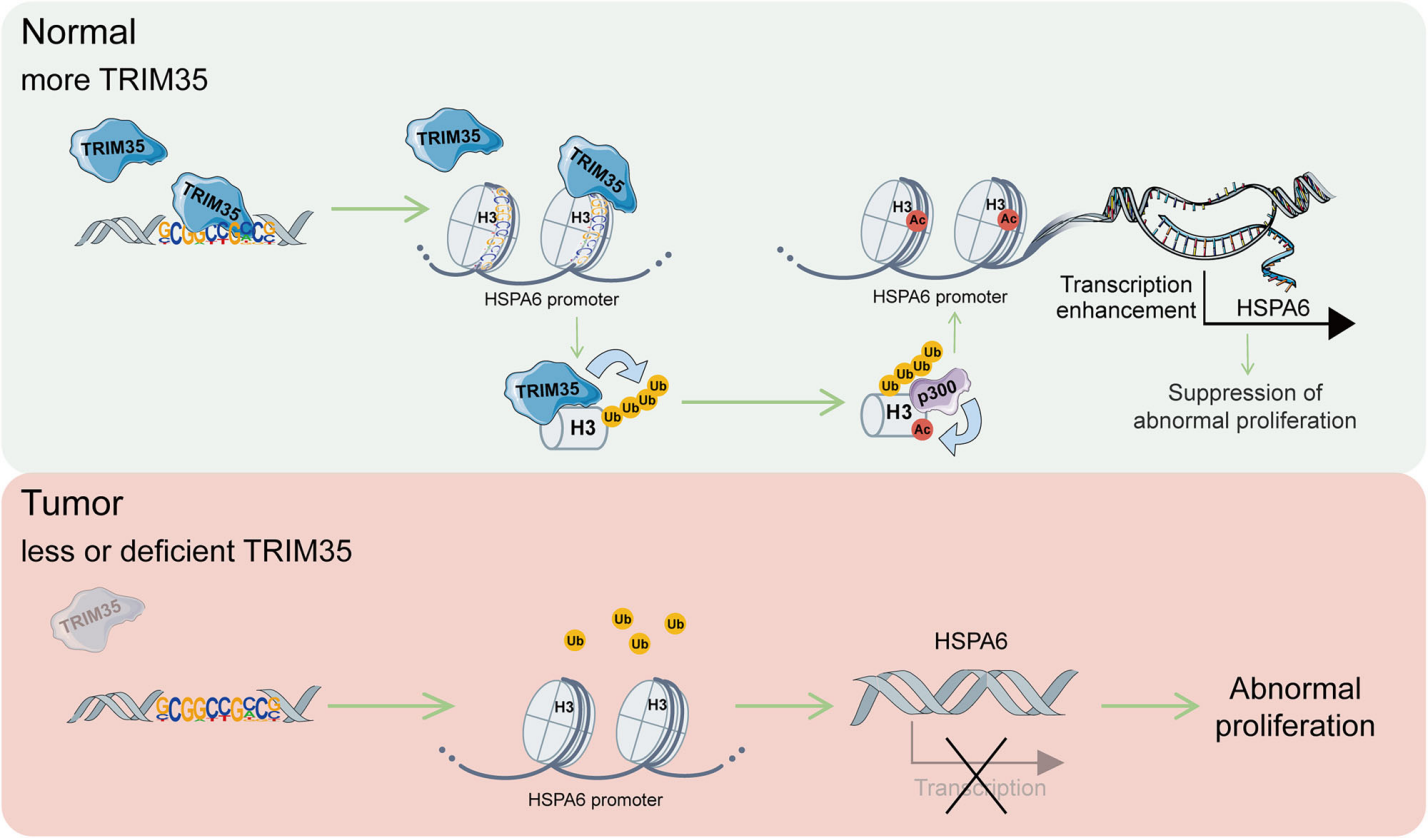
Mechanism diagram. TRIM35 binds to the HSPA6 promoter and polyubiquitinates H3, and subsequently the ubiquitination signal recruits p300 to acetylate H3K27, which increases the transcription of HSPA6 and consequently suppresses abnormal proliferation in breast cancer cells.

In conclusion, our study demonstrates that TRIM35 preferentially targets gene promoters, interact with histone H3, and catalyze its non-proteolytic polyubiquitination, which serves as a key signal for p300 recruitment, ultimately leading to H3K27 acetylation and transcriptional activation (Fig. 8). These findings significantly advances the understanding of TRIM35’s function in epigenetic regulation and its potential role as a therapeutic target in breast cancer. Future research should explore whether TRIM35 has similar functions in other types of cancer and whether it can be targeted to modulate tumor progression through its epigenetic regulatory activity.

## Materials and methods

### Cell lines and cell culture

Human breast cancer cell lines (MDA-MB-231, MCF7 and CAL51) were obtained from the Key Laboratory of Environment and Genes Related to Diseases at Xi’an Jiaotong University (Xi’an, China). MDA-MB-231 and MCF7 cells were cultured in Dulbecco’s Modified Eagle’s Medium (DMEM; Gibco) supplemented with 10% fetal bovine serum (FBS; Gibco) and 1% penicillin-streptomycin (PS; Solarbio), CAL51 cells were maintained under the same conditions but with 20% FBS supplementation.

The MCF10A cell line and its corresponding DMEM/F12 medium were purchased from Procell Life Science & Technology (Hubei, China). The DMEM/F12 medium was supplemented with 5% horse serum (HS), 20 ng/mL epidermal growth factor (EGF), 0.5 μg/mL hydrocortisone, 10 μg/mL insulin, 1% non-essential amino acids (NEAA), and 1% penicillin-streptomycin (PS).

All cell lines were maintained at 37 °C in a humidified incubator with 5% CO₂. All cells were regularly tested for *Mycoplasma* contamination and were negative.

For glucose depletion experiments, cells were transferred to Dulbecco’s Modified Eagle Medium, No Glucose (DMEM, Biosharp) supplemented with 10% FBS and 1% PS. After incubation in this medium for 4 h, cells were harvested for subsequent analysis.

### Immunohistochemical staining

All breast cancer and adjacent non-cancerous tissues were collected from Xianyang Hospital of Yan’an University in China, and the study was approved by the Ethics Committee of Xianyang Hospital, Yan’an University (No. YDXY-KY-2023-022). Immunohistochemical staining was performed according to the manufacturer’s protocol of the Biotin-Streptavidin HRP Detection Systems (PV-9001, ZSGB-BIO) and immunodetection was performed using the 3,3’-diaminobenzidine kit (DAB, ZSGB-BIO). The primary antibody was anti-TRIM35 (1:200 dilution, Abcam, ab272582, UK).

### RNA extraction, reverse transcription, and real-time PCR

Total cellular RNA was extracted using TRIzol reagent (GenStar, China) following the manufacturer’s instructions. Reverse transcription was performed using the Hifair® Ⅲ 1st Strand cDNA Synthesis SuperMix for qPCR (gDNA digester plus) (YESEN, China) according to the manufacturer’s protocol. Quantitative real-time PCR (qRT-PCR) was conducted using Hieff® qPCR SYBR Green Master Mix (No Rox) (YESEN, China). Relative gene expression levels were normalized to GAPDH and calculated using the 2^−ΔΔCt^ method. The primers employed in this study are detailed in Supplementary Table S1.

For RNA sequencing analysis, cell samples resuspended in TRIzol were sent to Biomarker Technologies for RNA extraction and sequencing.

### Western blotting

As previously described [52], proteins were extracted using RIPA buffer (Beyotime, China) supplemented with protease and phosphatase inhibitors. Protein concentrations were measured using the BCA Protein Assay Kit (Beyotime, China). Protein samples were separated on 6–12.5% SDS-PAGE gels and transferred onto PVDF membranes (Millipore, USA). Membranes were blocked with 5% skim milk, incubated with primary antibodies at 4 °C overnight, followed by secondary antibody incubation at room temperature for 1 h. Protein signals were detected using an ECL kit (Mishu, China) and visualized with the ChemiDoc MP Imaging System (BIO-RAD, # 12003154). The antibodies employed in this study are detailed in Supplementary Table S2. All original Western blots were detailed in Supplementary File—Original Western blots.

### siRNA synthesis, plasmid construction, cell transfection and stable cell line construction

HSPA6 and p300 siRNAs were synthesized by GenePharma (Shanghai, China), with a non-sense siRNA used as a negative control. Ub-V5 and mutant plasmids (Ub-K48R-V5 and Ub-K63R-V5) were obtained from Genechem (Shanghai, China). The siRNA sequences employed in this study are detailed in Supplementary Table S3.

The coding sequences (CDS) of TRIM35, HSPA6, and H3 were amplified from cDNA derived from MCF10A cells and cloned into GV141 vectors (for TRIM35 and HSPA6) and GV219 vectors (for H3), both purchased from Genechem (Shanghai, China). TRIM35 domain deletion constructs were generated using overlap PCR and cloned into GV141 vectors. H3 mutant plasmids were constructed following the manufacturer’s instructions for the Fast Mutagenesis System kit (TransGen Biotech, China).

The pLenti-TRIM35 plasmid was constructed by cloning the TRIM35 CDS into the pLV-C-GFPSpark vector (Sino Biologica, Beijing). To generate lentiviral particles, GFP-tagged pLenti-TRIM35, control plasmid, and packaging plasmids were co-transfected into HEK-293FT cells. The lentivirus-containing supernatant was collected at 48 and 72 h post-transfection, filtered, and used to infect cancer cells for 24 h. Following infection, GFP-positive cells were sorted using flow cytometry and subsequently cultured to establish stable cell lines.

All constructed plasmids were verified via DNA sequencing. Cell transfection was performed using jetPRIME™ reagent (Polyplus-Transfection, France) according to the manufacturer’s protocol.

### MTT and colony formation assay

Cells (3–5 × 10^3^) were seeded into 96-well plates and transfected with plasmids or siRNA when cell confluency reached 40–60% the following day. At 24, 48 and 72 h post-transfection, 10 µL of MTT solution (5 mg/mL) was added to each well. After 4 h of incubation at 37 °C, the supernatant was removed, and 150 µL of dimethyl sulfoxide (DMSO) was added to each well. Absorbance was measured at 492 nm using a microplate reader (FLUOstar OPTIMA, BMG, Germany).

Cells (1–1.5 × 10^5^) were seeded into 12-well plates and transfected with plasmids or siRNAs when cell confluency reached 40–60% the following day. Six hours post-transfection, cells were digested, resuspended, and reseeded into new 12-well plates at 400–600 cells per well. The cells were then cultured for 2 weeks at 37 °C. After incubation, colonies were fixed with 4% paraformaldehyde for 30 min at room temperature and stained with 1% crystal violet for 30 min at room temperature. Colony images were captured using Quantity One® software (version 4.6.2, Bio-Rad Laboratories, Inc.), and ImageJ was used to quantify the colony number.

### Cell cycle and apoptosis detection

Cells were collected and washed twice with PBS. For cell cycle detection, cells were then fixed with 70% ethanol at 4 °C overnight. The following day, cells were washed and resuspended in 150 μL RNase A (100 μg/mL) for 5 min at room temperature, followed by incubation with 150 µL propidium iodide (PI, 50 µg/mL) on ice for 15 min before analysis.

For apoptosis detection, cells were then resuspended in 1 × binding buffer and stained using the Annexin-V-FITC/PI Apoptosis Detection kit (Yeasen Biotechnology, Shanghai, China) according to the manufacturer’s protocol.

Both cell cycle and apoptosis analyses were performed using flow cytometry (FACSCalibur, BD Biosciences, USA).

### Wound-healing assay

At 6 h post-transfection, a 10 µL pipette tip was used to create a linear wound in the cell monolayer. Wound closure was monitored and images were captured at 0, 24, 48, and 72 h using a microscope (Nikon, Japan).

### Tumorigenic assay in nude mice

Four-week-old female BALB/c nude mice were purchased from the Laboratory Animal Center, Xi’an Jiaotong University Health Science Center, and housed under specific-pathogen-free (SPF) conditions. All experimental procedures were conducted in accordance with the standards of the Institutional Animal Care and Use Committee of Xi’an Jiaotong University (No. XJTUAE2024-2672).

The mice were randomly divided into two groups. The pLenti-ctrl and pLenti-TRIM35 cells (2 × 10⁶) were resuspended in 100 µL PBS and injected bilaterally into the inguinal region of nude mice (*n* = 3/group). Tumor growth was monitored starting 8 days post-injection, with measurements taken every 4 days thereafter. The volume of the xenografts was calculated using the formula: V = (L × W^2^)/2 (W: shortest dimension and L: longest dimension). After 28 days, mice were euthanized under deep anesthesia, and xenografts were surgically excised and stored for subsequent experiments.

### Immunofluorescence assay

Cells were fixed with 4% paraformaldehyde for 30 min at room temperature, then permeabilized with PBST (0.1% Triton X-100 in PBS) for 15 min. Following permeabilization, cells were blocked with 5% BSA for 1 h at room temperature. Cells were then incubated with primary antibodies overnight at 4 °C. The following day, cells were incubated with fluorescent secondary antibodies and DAPI in the dark at room temperature. Fluorescence imaging was performed using a fluorescence microscope (Leica TCS SP8 DIVE, Germany).

### Co-Immunoprecipitation assay (Co-IP)

Cells were lysed using the Mammalian Cell Lysis Reagent (Pioneer Biotechnology, China) supplemented with protease and phosphatase inhibitors. The lysate was then gently mixed by rotation for 1 h at 4 °C, followed by centrifugation at 14,000 rpm for 20 min. The supernatant lysate was incubated with primary antibodies at 4 °C overnight with gentle mixing. The following day, magnetic beads (Invitrogen, CA, USA) were washed twice with PBS and added to lysate. The mixture was incubated at 4 °C for 4 h with gentle rotation. After incubation, the magnetic beads were collected, and the bound proteins were eluted for western blot analysis.

### Chromatin immunoprecipitation assay (ChIP)

Cells were cross-linked with 1% formaldehyde for 15 min, and the reaction was quenched by adding 125 mM glycine for 30 min at room temperature. Chromatin was then sheared into 200–500 bp fragments via sonication. Primary antibodies were added, and the mixture was incubated overnight at 4 °C. The next day, magnetic beads (Invitrogen, CA, USA) were introduced, followed by an additional 4 h incubation at 4 °C with gentle mixing. After incubation, the magnetic beads were collected, and protein-DNA complexes were eluted. The complexes were then de-crosslinked at 65 °C and digested with RNase A and proteinase K. DNA was subsequently purified using the phenol-chloroform method and analyzed via RT-PCR. The purified DNA was sent to Novogene Co., Ltd. for sequencing.

### Bioinformatics analysis

The Cancer Genome Atlas (TCGA) and Genotype-Tissue Expression (GTEx) data were downloaded from the UCSC XENA platform (https://xena.ucsc.edu) and analyzed using the R packages, including ggstatsplot, survival and survminer. Gene set enrichment analysis (GSEA) was performed using the clusterProfiler R package.

ChIP-seq data analysis: ChIP-seq peaks were annotated using the ChIPSeeker R package. Sequencing data were processed and mapped using deepTools, while HOMER (http://homer.ucsd.edu/homer/) was utilized to identify binding motifs.

RNA-seq data analysis: sequencing reads were mapped to the reference genome using Hisat2, and differential gene expression was analyzed using DESeq2. Differentially expressed genes (DEGs) were identified based on the following criteria: *p* value < 0.05 and fold-change ≥2.

Gene Ontology (GO) and Kyoto Encyclopedia of Genes and Genomes (KEGG) analyses were performed and plotted by https://www.bioinformatics.com.cn, an online platform for data analysis and visualization [53].

### Statistical analysis

All experiments were repeated independently three times, and statistical analysis was performed using SPSS 24.0. The *t*-test was used to compare two groups, while one-way analysis of variance (ANOVA) was applied for multiple comparisons among more than two groups. Data are presented as the mean ± standard deviation (SD). *p < 0.05 was considered statistically significant.

## Supplementary information

Supplementary Figure

Supplementary Table

Original western blots

## Data Availability

The data supporting the findings of this study can be obtained from the corresponding author upon reasonable request.
