# Supplementary material for: TRIM35, a novel DNA-binding protein, epigenetically modifies H3 to promote HSPA6 transcription and suppress breast cancer progression
Source: Cell Death Discov. 2025 Oct 24;11:479. doi: 10.1038/s41420-025-02770-9 (PMC12552751; doi:10.1038/s41420-025-02770-9)
Supplement: Supplementary file 1 — Supplementary Figure [file 41420_2025_2770_MOESM1_ESM.docx]

**Supplementary Figure:**


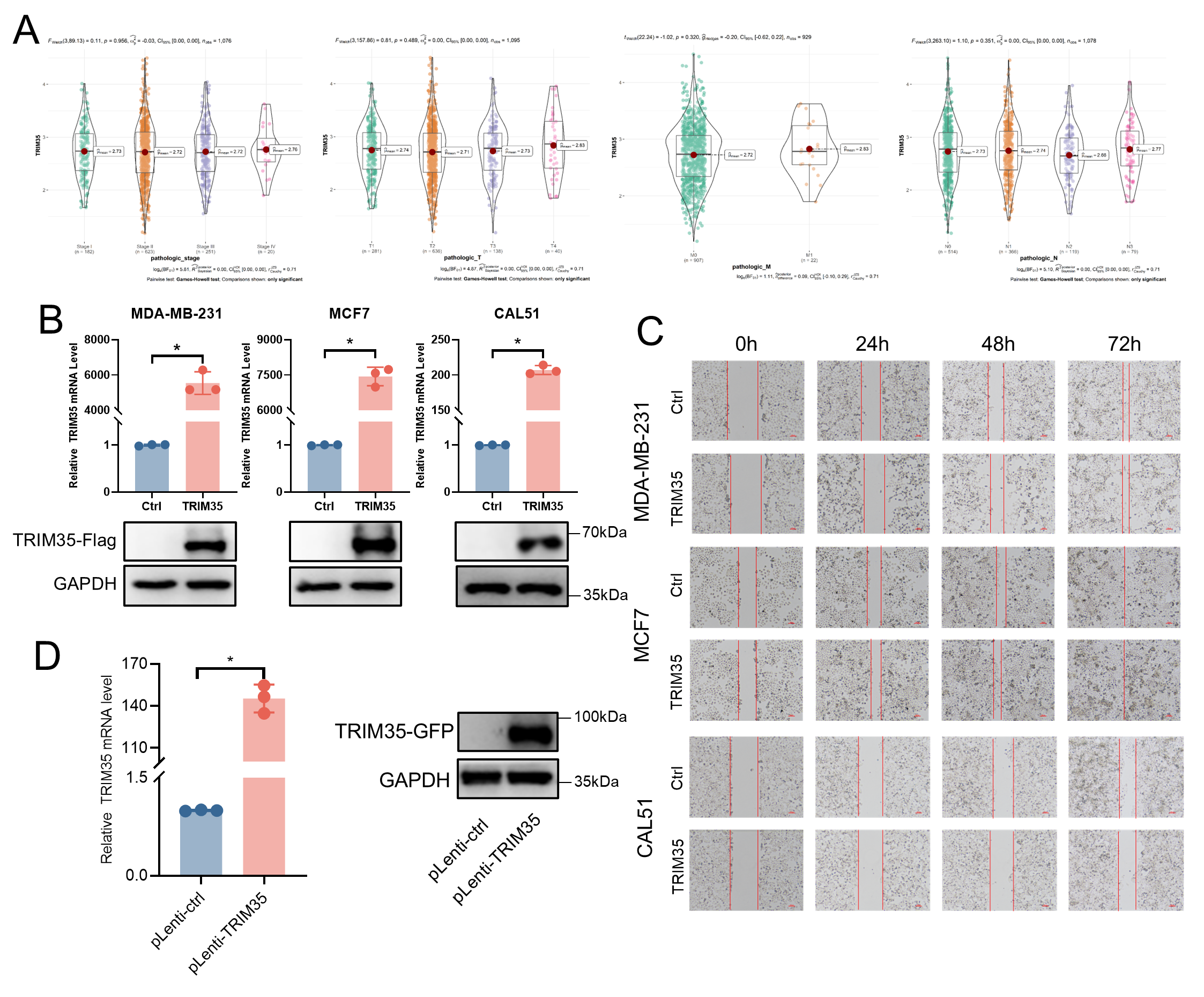


**Figure S1: TRIM35 was not associated with tumor stage, metastasis, or migration of breast cancer cells.** A. Analysis of the correlation between TRIM35 expression and tumor stage or metastasis using TCGA and GTEx databases. B. qRT-PCR and western blotting to assess TRIM35 overexpression efficiency in breast cancer cells. C. Wound-healing assay was used to detect the effect of TRIM35 overexpression on breast cancer cell migration. D. qRT-PCR and western blotting were utilized to detect the efficiency of TRIM35 overexpression in stable cell lines. Data are presented as mean ± SD (*n* = 3), * means *P* < 0.05.


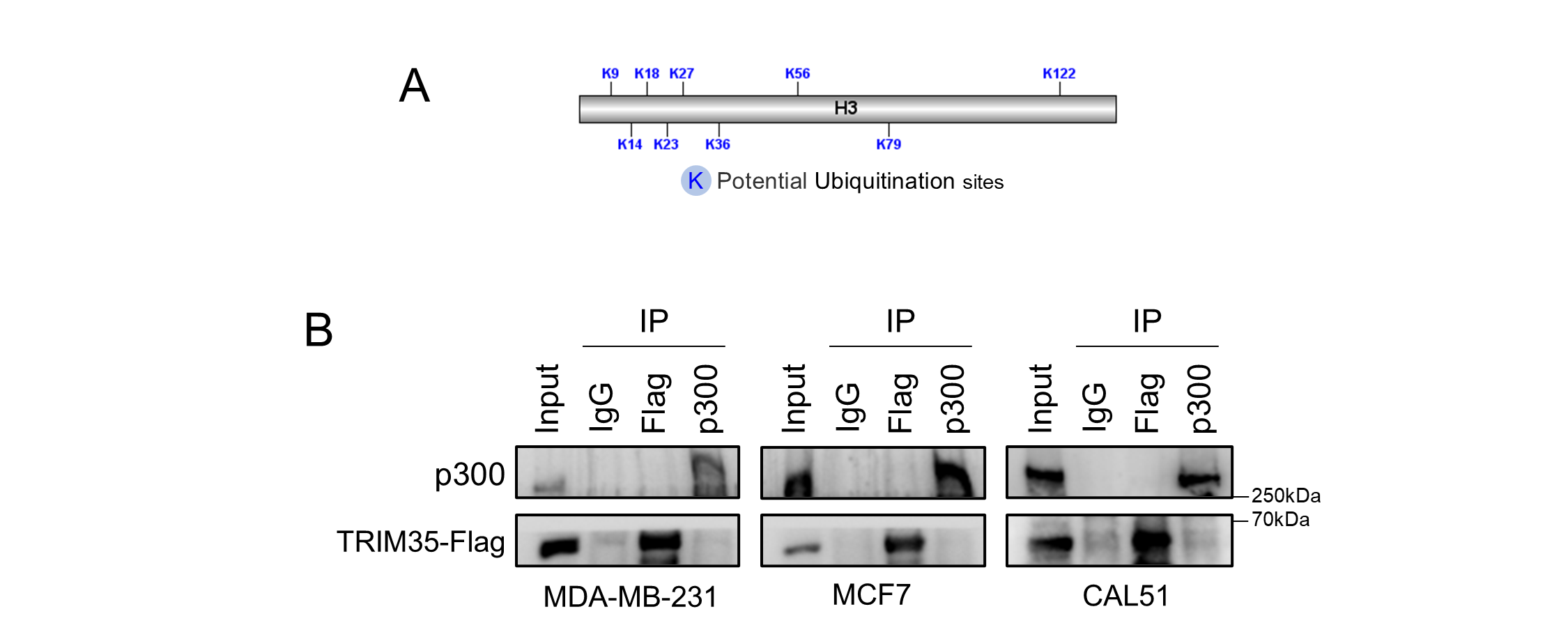


**Figure S2: TRIM35 fails to interact with p300.** A. Schematic diagram illustrating potential ubiquitination sites on H3. B. Co-IP and western blotting were used to detect the interactions between TRIM35 and p300.

**
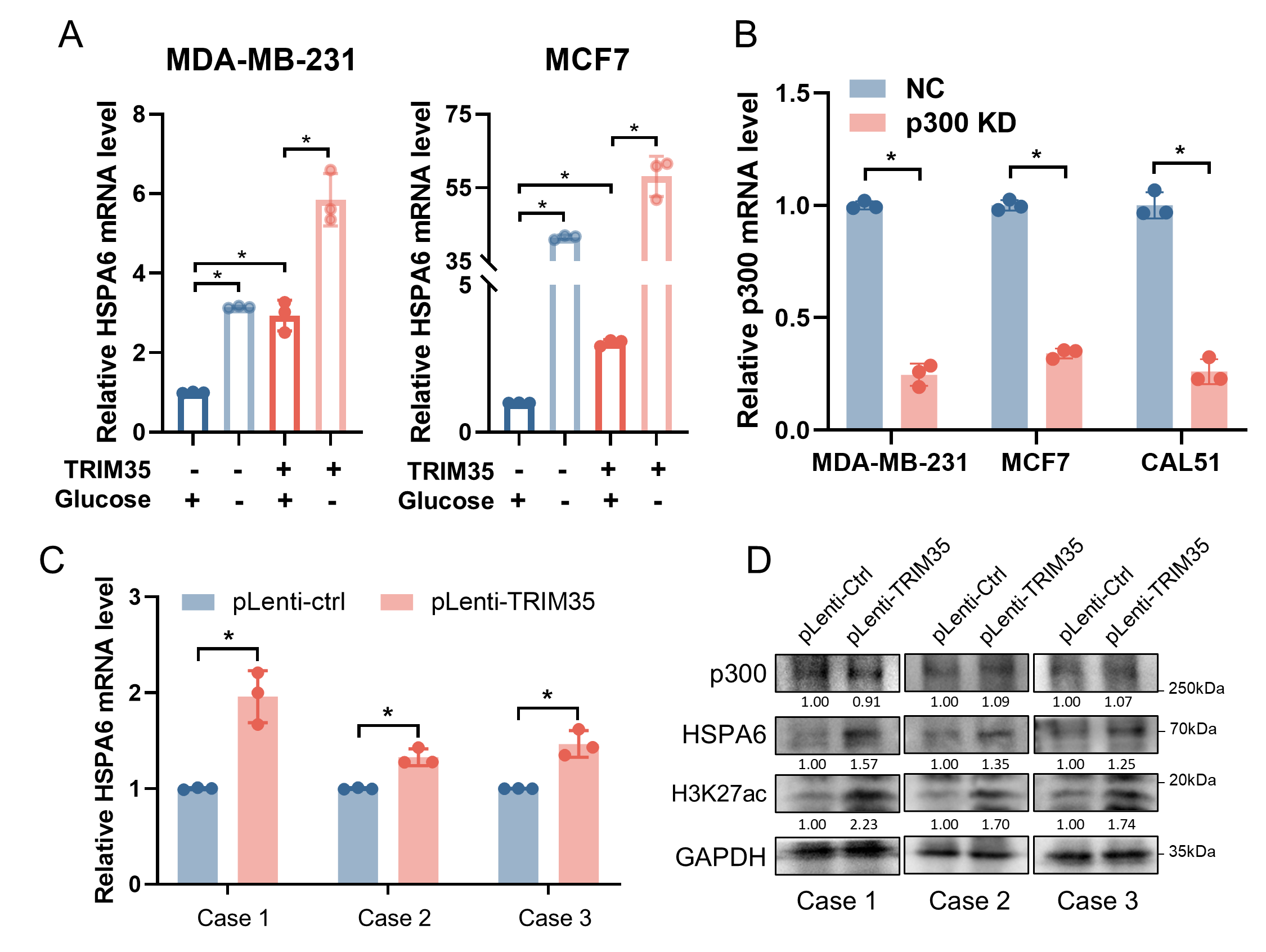
**

**Figure S3: Analysis of glucose-induced HSPA6 mRNA levels, p300 knockdown efficiency, and TRIM35-mediated regulation of HSPA6 in xenograft tumors.** A. qRT-PCR was employed to detect the effect of glucose removal on HSPA6 expression. B. qRT-PCR was used to detect the efficiency of p300 knockdown in breast cancer cells. qRT-PCR (C) and western blotting (D) were performed to detect HSPA6 mRNA levels and the protein expression of key molecules of HSPA6 regulated by TRIM35 in tumor tissues from the TRIM35-overexpression xenograft models. Data are presented as mean ± SD (*n* = 3), * means *P* < 0.05.

**
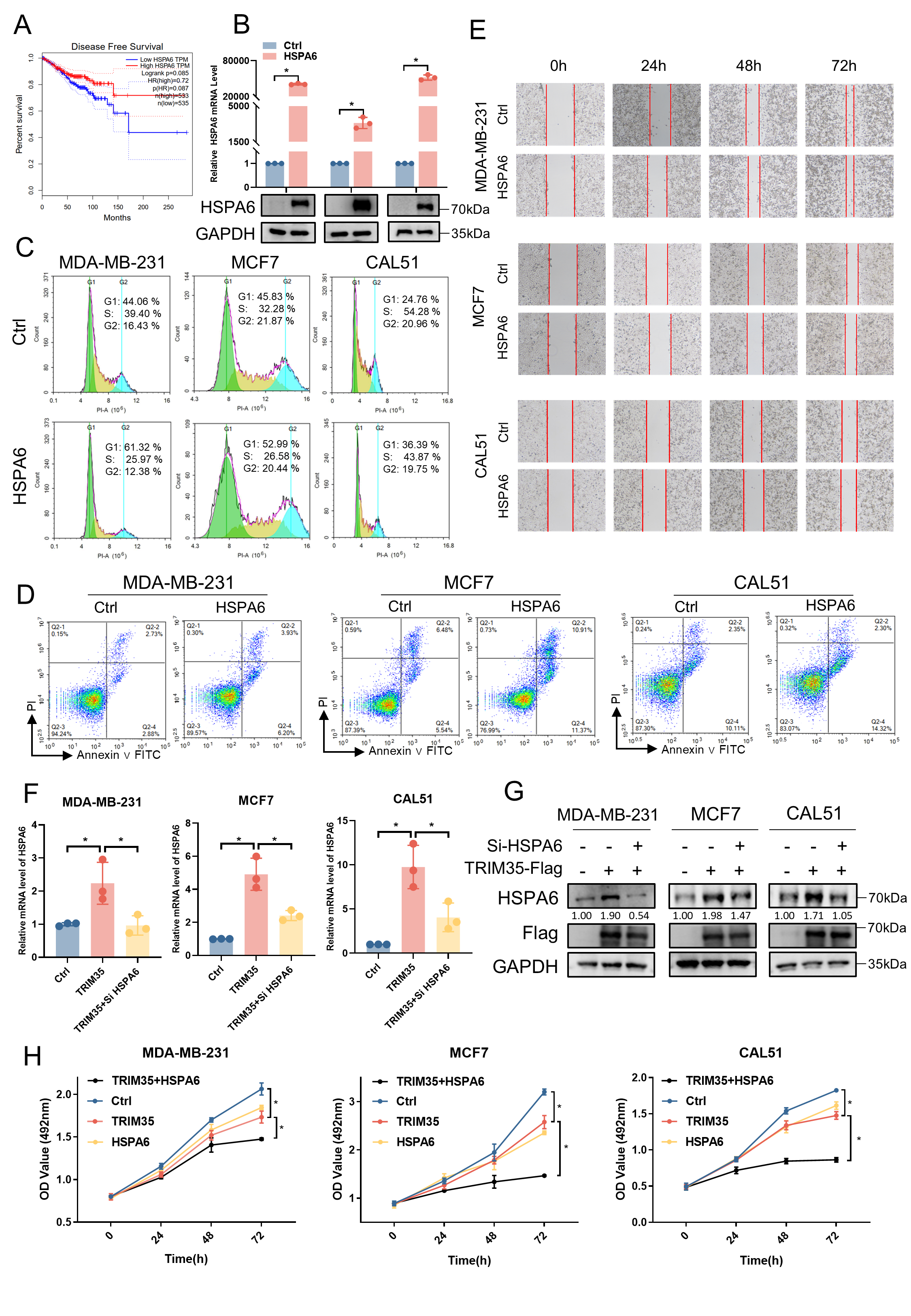
**

**Figure S4: TRIM35 regulates HSPA6 to suppress breast cancer cell progression.** A. Kaplan-Meier survival analysis of HSPA6 expression in breast cancer patients from the GEPIA2 database. B. qRT-PCR and western blotting were employed to determine the efficiency of HSPA6 overexpression in breast cancer cells. Flow cytometry was utilized to detect the impact of HSPA6 overexpression on cell cycle (C) and apoptosis (D) in breast cancer cells. E. Wound-healing assay was performed to detect the effect of HSPA6 overexpression on breast cancer cell migration. qRT-PCR (F) and western blotting (G) were used to detect the efficiency of HSPA6 knockdown in breast cancer cells. H. MTT assay was used to assess the synergistic effect of HSPA6 on TRIM35-mediated tumor suppression. Statistical comparisons were performed at 72 h. Data are presented as mean ± SD (*n* = 3), * means *P* < 0.05.
