# Supplementary material for: TRIM35, a novel DNA-binding protein, epigenetically modifies H3 to promote HSPA6 transcription and suppress breast cancer progression
Source: Cell Death Discov. 2025 Oct 24;11:479. doi: 10.1038/s41420-025-02770-9 (PMC12552751; doi:10.1038/s41420-025-02770-9)
Supplement: Supplementary file 2 — Supplementary Table [file 41420_2025_2770_MOESM2_ESM.docx]

**Supplementary Table:**

**Table S1: The primers employed in this study**

| **Name** | **Sequence** |
| --- | --- |
| TRIM35 | F:CCATCGCCAAGCACAATC |
|  | R:CGTCCTCCTTCATCTCCATCT |
| HSPA6 | F:GCTGAGCAAGATGAAGGAGACG |
|  | R:GATGATCCGCAACACGTTGAGC |
| TMEM121 | F:AGCACATAGCGCCGCAGAAGAT |
|  | R:CGTTTTTGCCGACGAAGATGGC |
| DOCK2 | F:TGAAGCTGGACCACGAGGTAGA |
|  | R:GCCTTTGACCAGGTTCACGAAG |
| GFRA3 | F:CCTACCTTTGCTGTGATGGCAC |
|  | R:CTACCATAGGCTCAGGAGCAGA |
| STBD1 | F:GGCAGGAGCTGGTCACCAAAC |
|  | R:TGTCACAGACTTCCCTGGAAGG |
| PRR18 | F:CCAGCGAGGACCACCTATGCG |
|  | R:TGACGCTTCTGGATGACCAGGA |
| GRAMD2A | F:CCAGGAAGCCTCCAATGTCTGA |
|  | R:GGAGAGCAGTTCGGCATCTTCT |
| p300 | F:GATGACCCTTCCCAGCCTCAAA |
|  | R:GCCAGATGATCTCATGGTGAAGG |
| GAPDH | F:GGAGCGAGATCCCTCCAAAAT |
|  | R:GGCTGTTGTCATACTTCTCATGG |
| ChIP-qPCR-HSPA6-site 1 | F:GAGGCGGGCTGGCCTGGCGTAG |
|  | R:GCCCGGGCTGACTCAGCCGGGT |
| ChIP-qPCR-HSPA6-site 2 | F:CCCGGGGTCAGCGCCGCGCCGC |
|  | R:CCCGACCCCCGCCGCCGCGAAC |

**Table S2: The antibodies employed in this study**

| **Antibody** | **Company** | **Catalog No** | **Notes** |
| --- | --- | --- | --- |
| Bax | proteintech | 50599-2-Ig |  |
| Bcl-2 | Cell Signaling | 15071S |  |
| Bcl-xl | proteintech | 10783-1-AP |  |
| CDK2 | HUABIO | ET1602-6 |  |
| CDK4 | proteintech | 11026-1-AP |  |
| Cleaved PARP | Cell Signaling | 5625S |  |
| Cyclin A2 | proteintech | 18202-1-AP |  |
| Cyclin D1 | proteintech | 60186-1-Ig |  |
| Flag | Abconal | AE092 |  |
| GAPDH | Abways | AB0038 |  |
| H3K4-mono methyl | Abcam | ab8895 |  |
| H3K4-tri methyl | Abcam | ab8580 |  |
| His | proteintech | 66005-1-Ig |  |
| Histone H3 | Abconal | A2348 | for western blotting and CoIP |
| Histone H3 | ZENBIO | 250011 | for IF |
| Histone H3 (acetyl K27) | HUABIO | A6D6 |  |
| HSPA6 | Santa Cruz | sc-374589 |  |
| Lamin B1 | proteintech | 12987-1-AP |  |
| p300 | Abcam | ab14984 |  |
| PARP | proteintech | 13371-1-AP |  |
| POLR2A | Abconal | A11181 |  |
| TRIM35 | Abcam | ab272582 |  |
| Ubiquitin | Santa Cruz | sc-166553 |  |
| V5-tag | proteintech | 14440-1-AP |  |

**Table S3: The siRNAs employed in this study**

| **Name** | **Sequence** |
| --- | --- |
| si NC | sense: TTCTCCGAACGTGTCACGT |
|  | antisense: ACGTGACACGTTCGGAGAA |
| si p300 | sense: CAATTCCGAGACATCTTGAGA |
|  | antisense: TCTCAAGATGTCTCGGAATTG |
| si HSPA6 | sense: CCACTTCATGGAAGAATTC |
|  | antisense: GAATTCTTCCATGAAGTGG |
