# Supplementary material for: TRIM35, a novel DNA-binding protein, epigenetically modifies H3 to promote HSPA6 transcription and suppress breast cancer progression
Source: Cell Death Discov. 2025 Oct 24;11:479. doi: 10.1038/s41420-025-02770-9 (PMC12552751; doi:10.1038/s41420-025-02770-9)

Fig. 1D

TRIM35

GAPDH

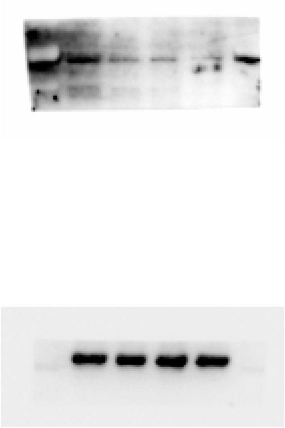

Fig. 2D

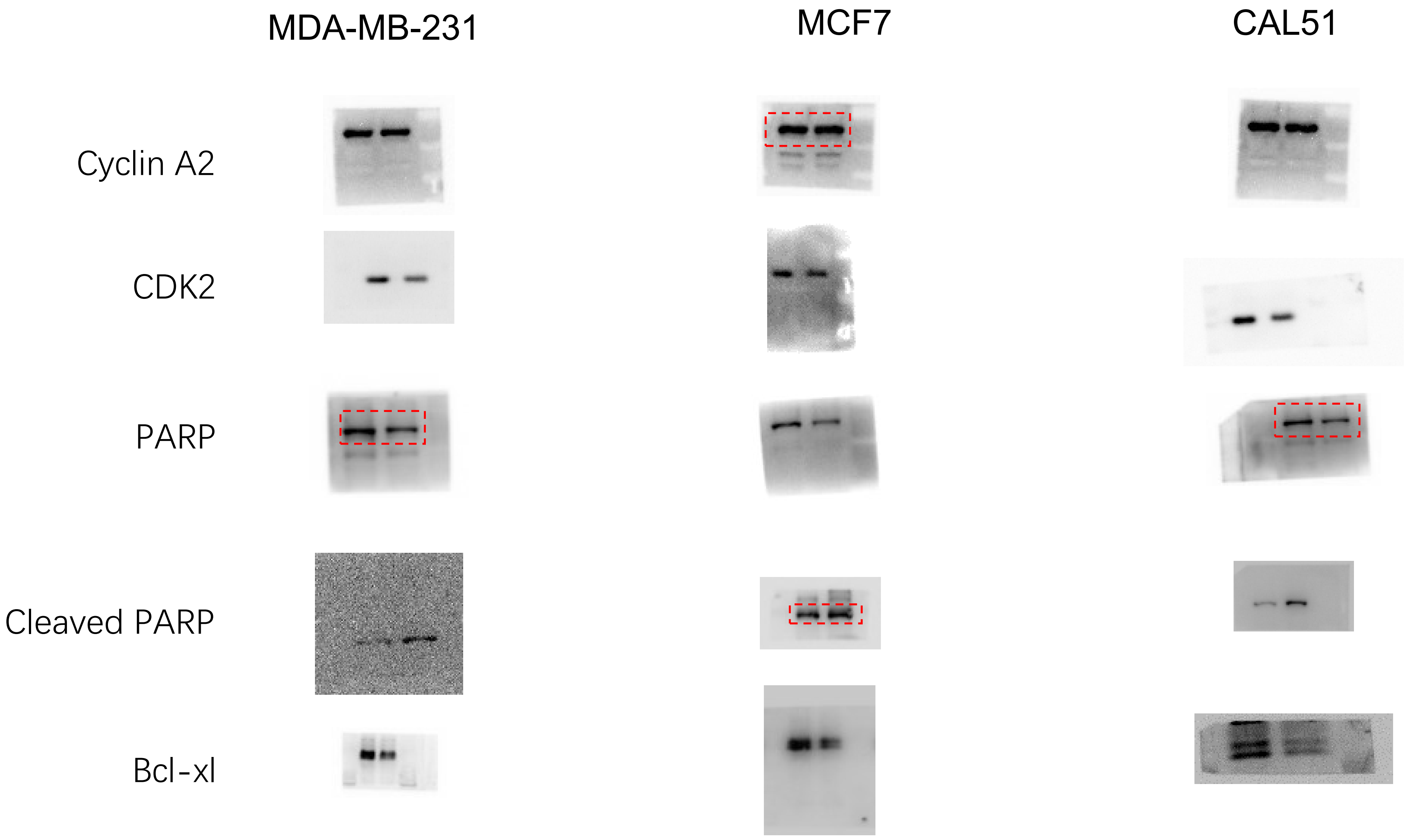

Fig. 2D

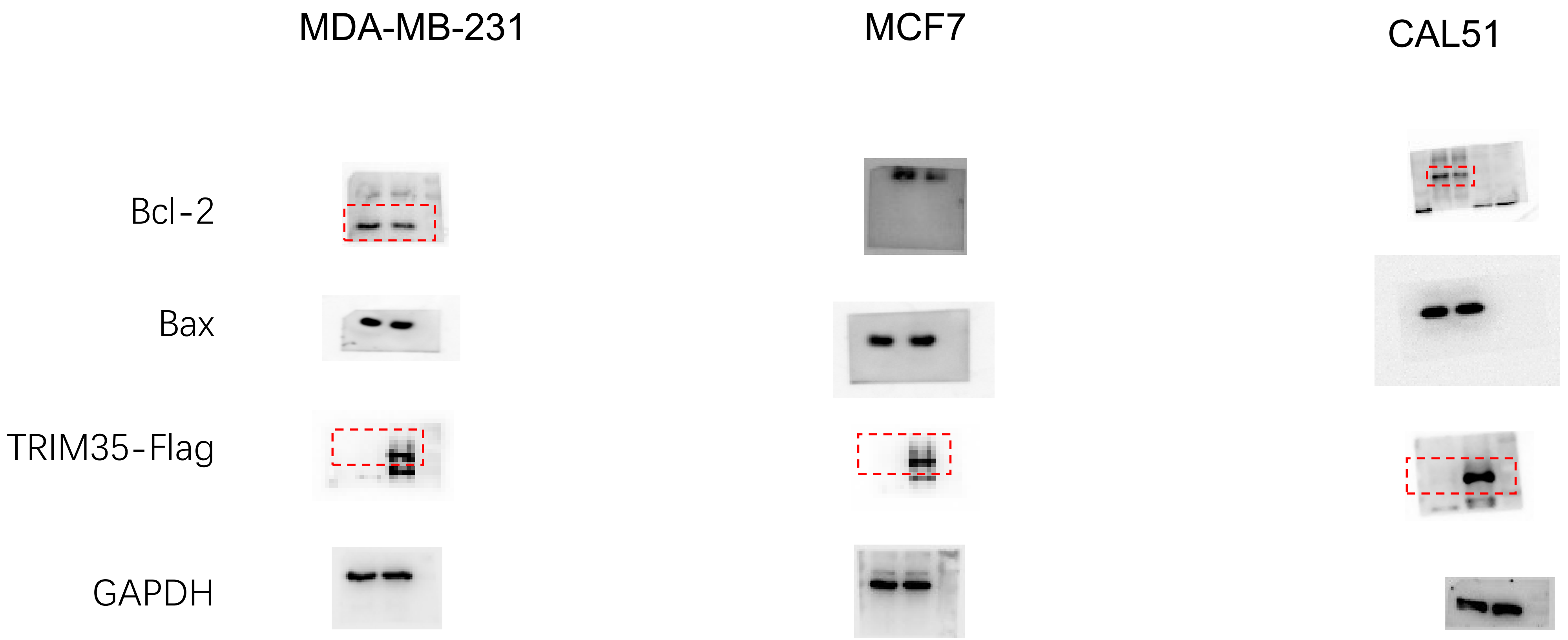

Fig. 2G

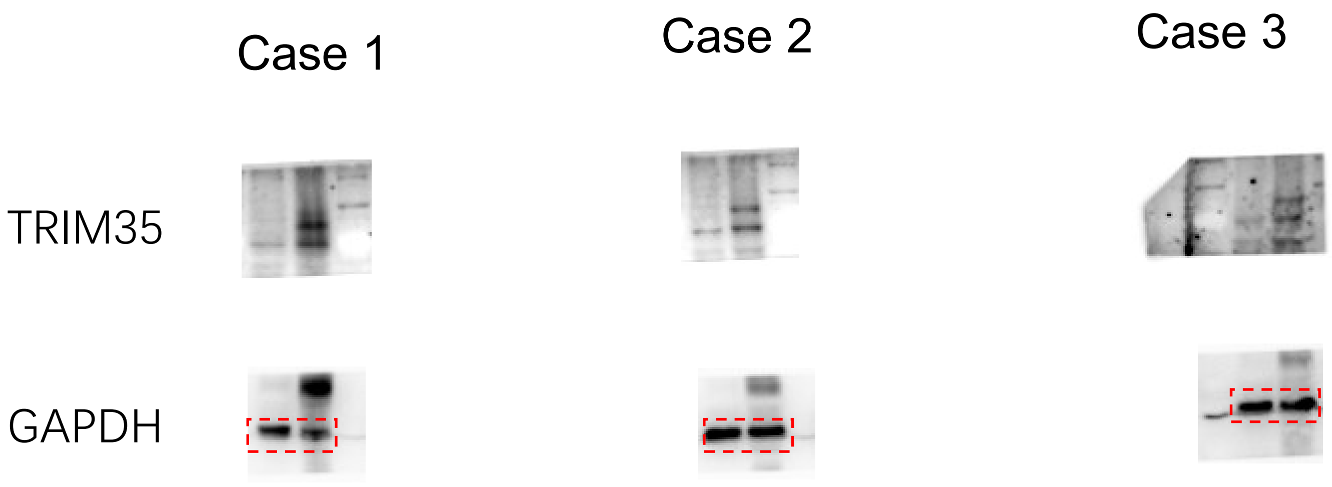

Fig. S1B

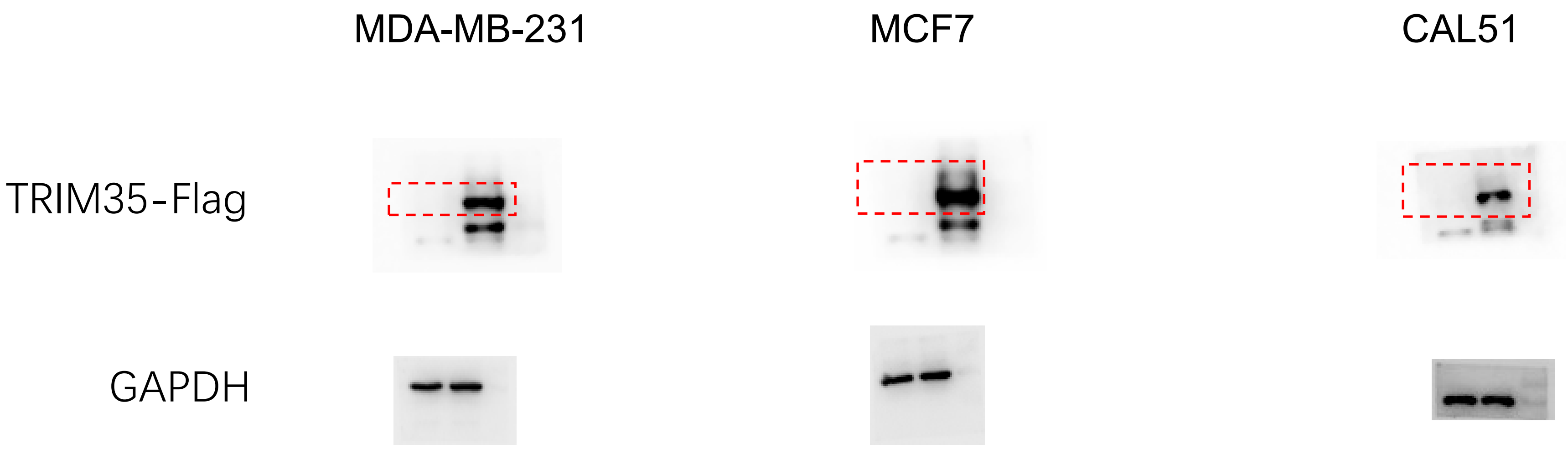

Fig. S1D

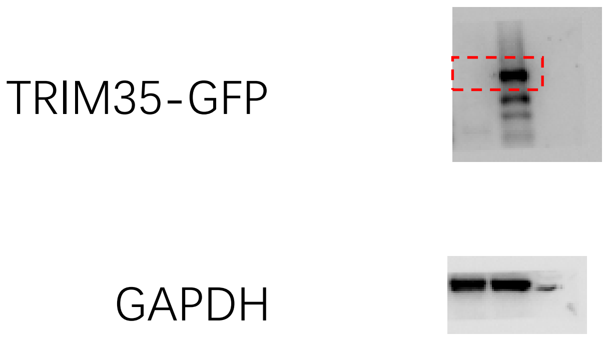

Fig. 3F

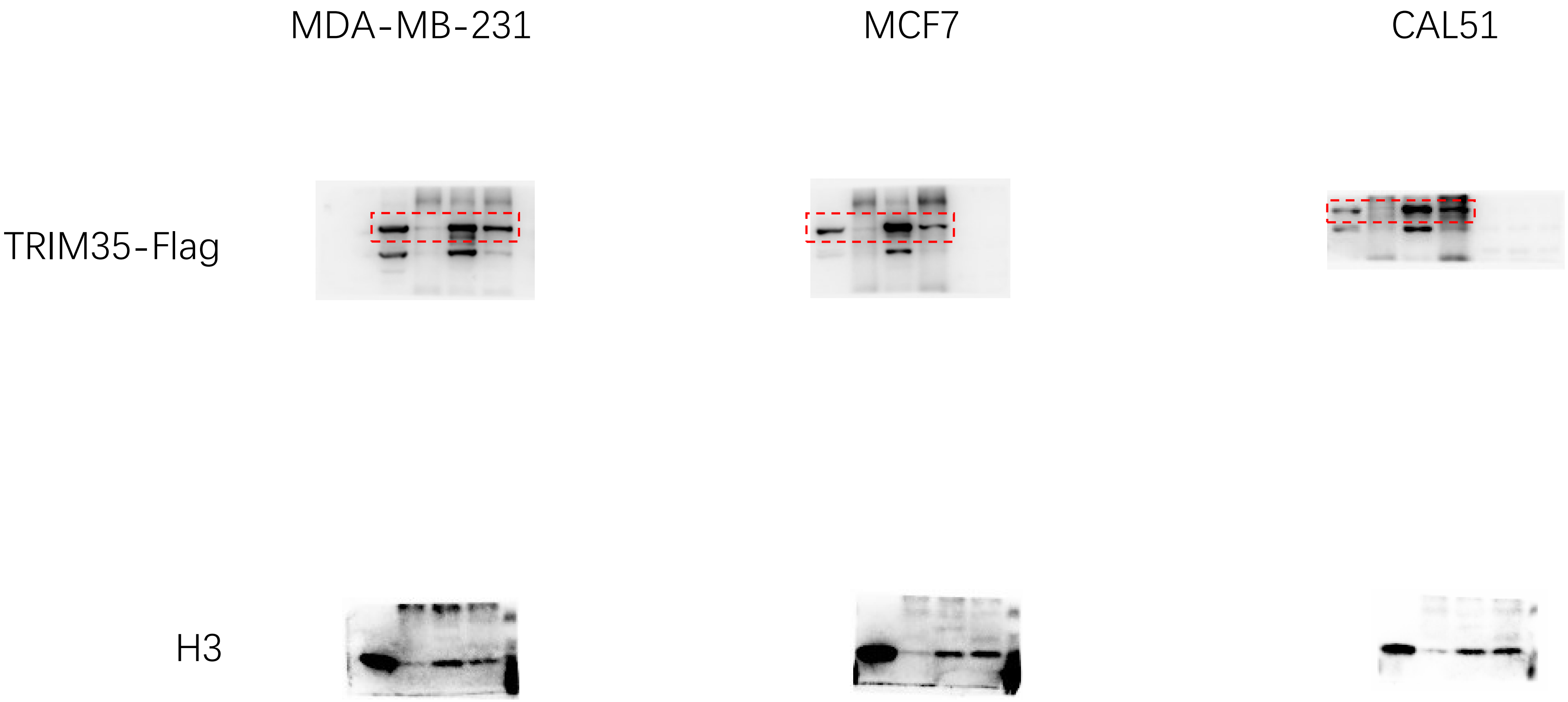

Fig. 3H

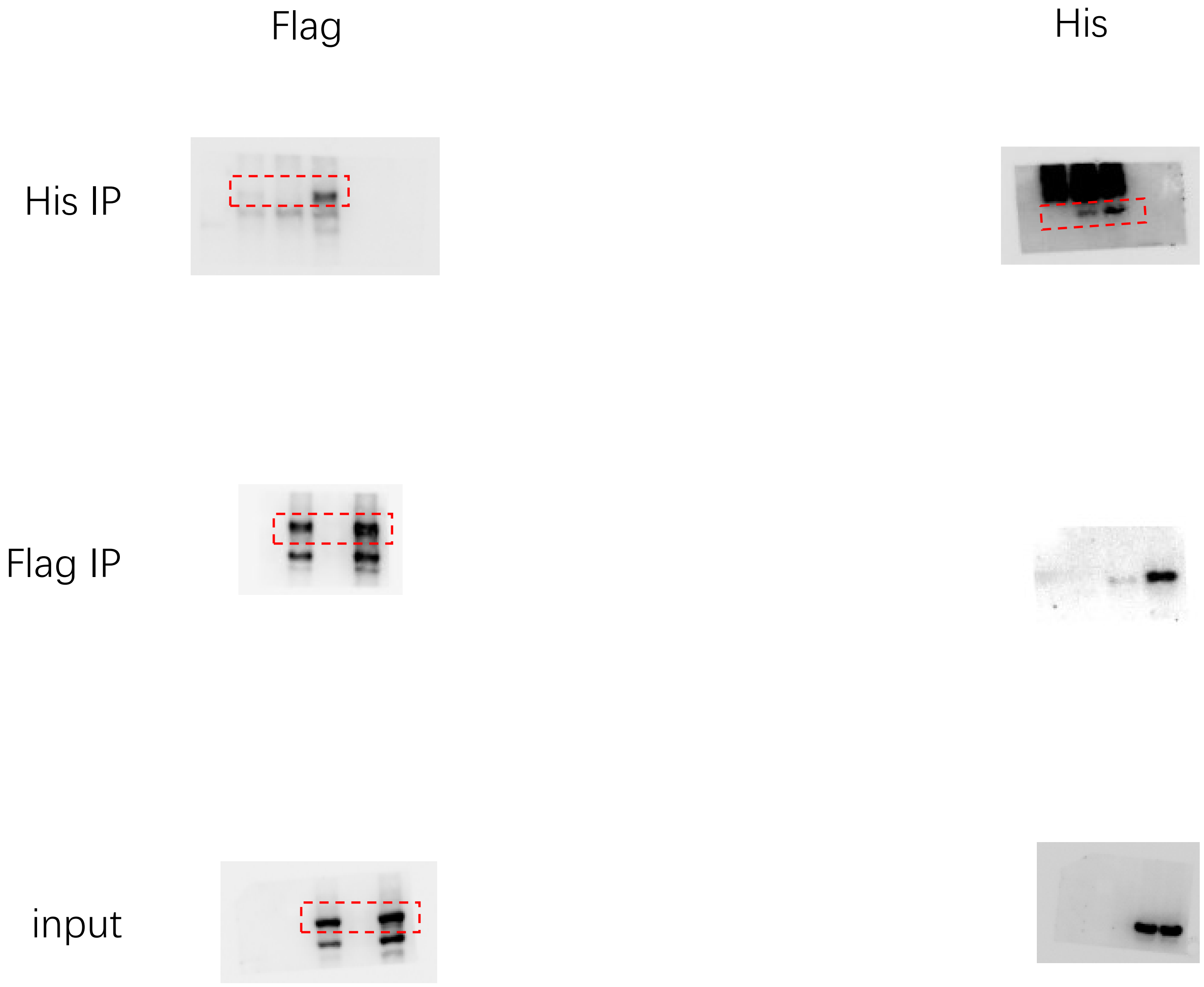

Fig. 3I

Flag | His

Flag IP

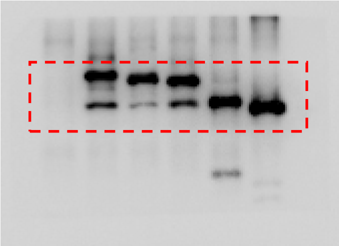

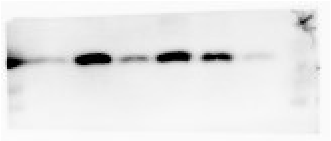

input

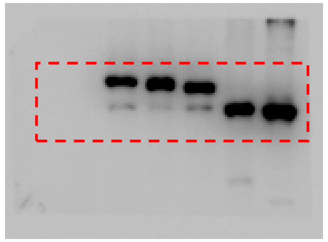

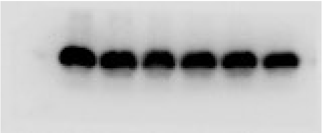

Fig. 4A

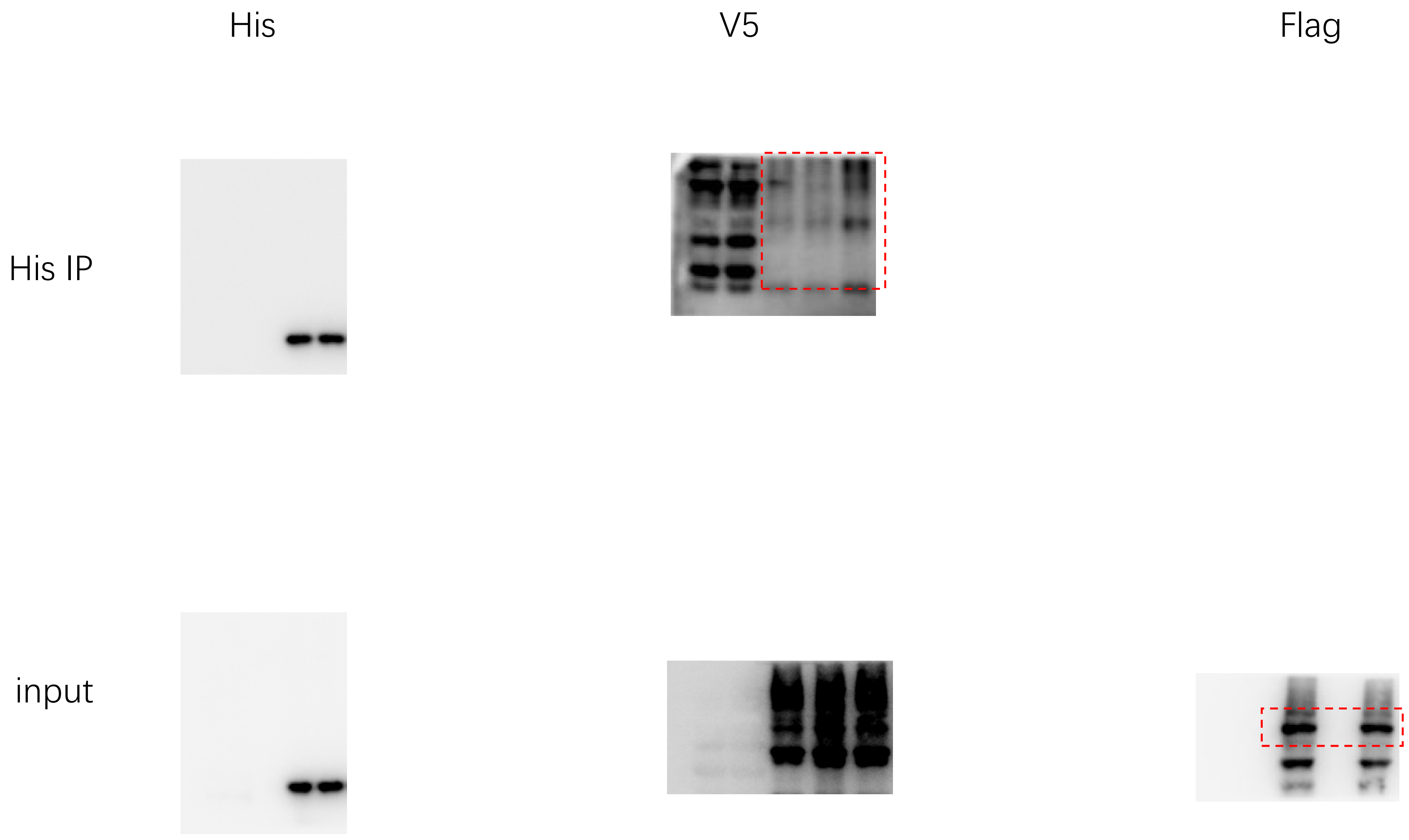

Fig. 4B

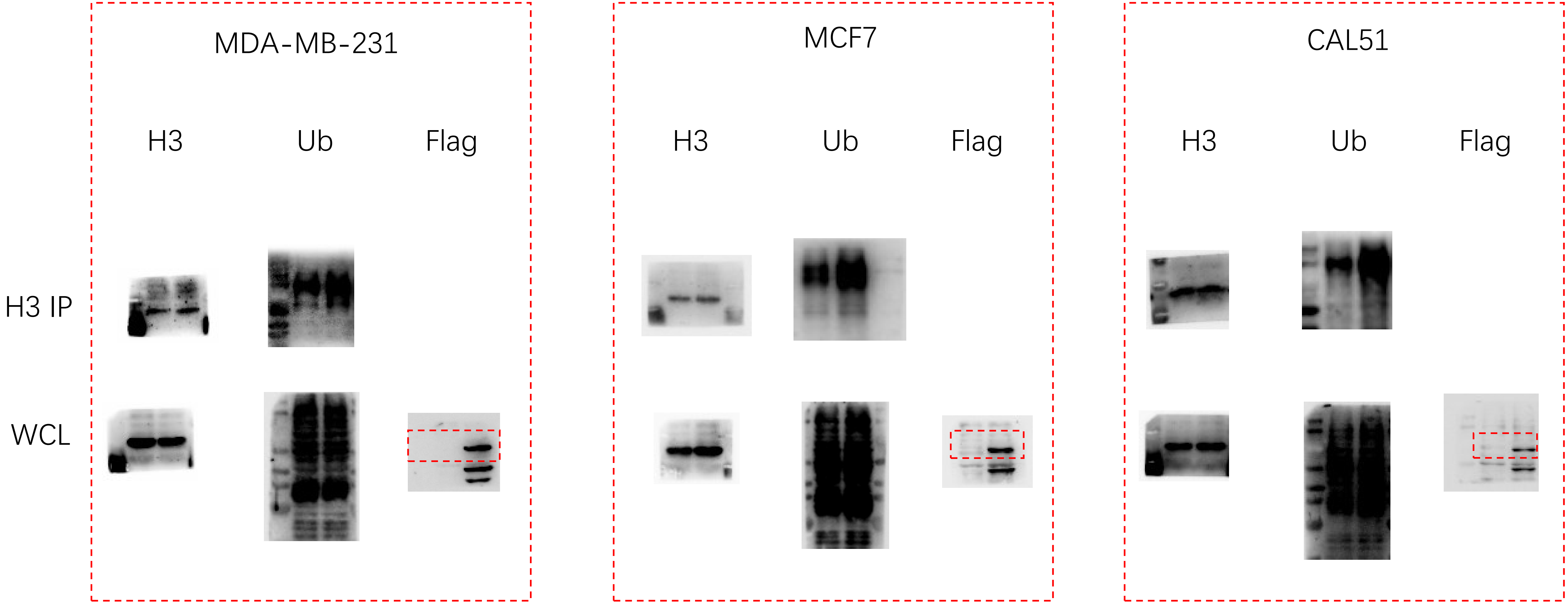

Fig. 4C

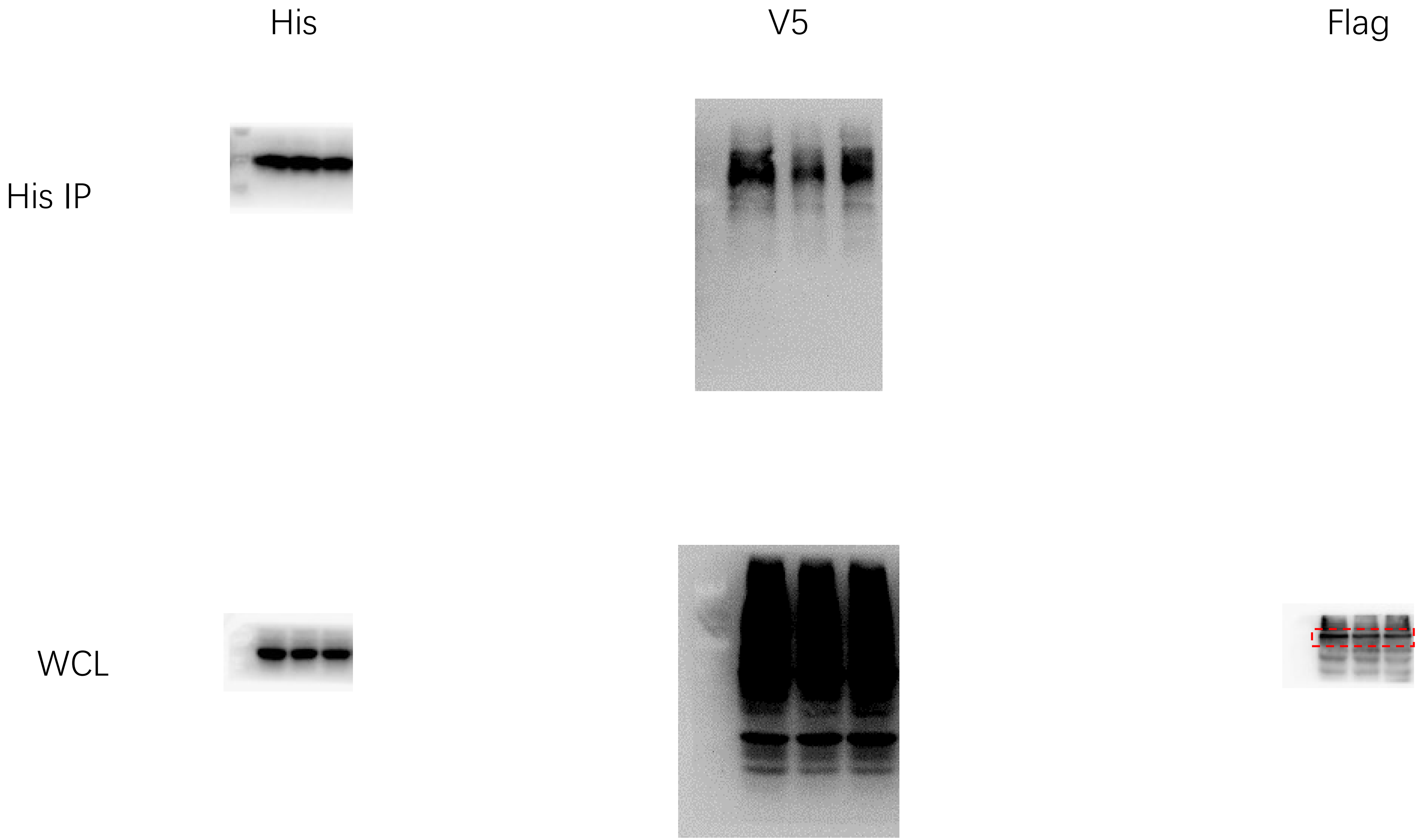

Fig. 4D

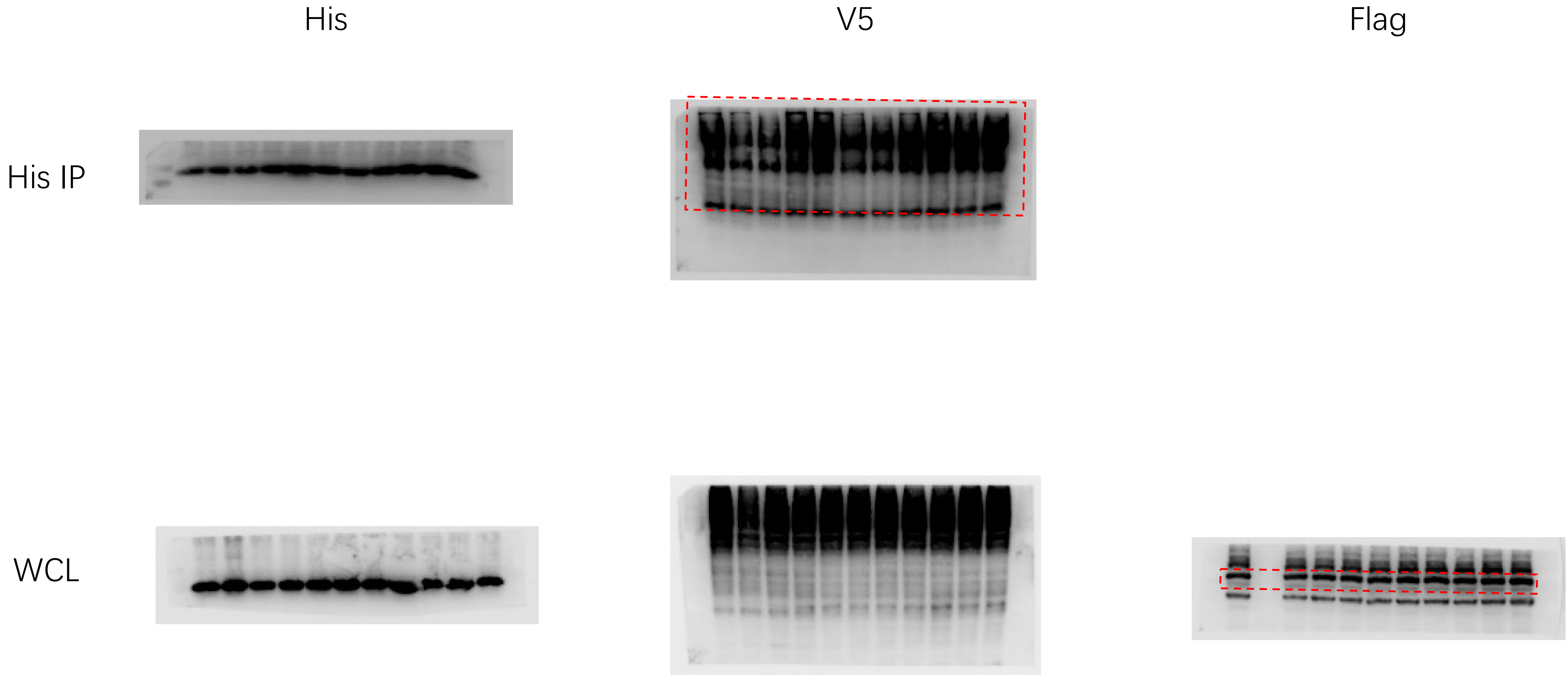

Fig. 4E

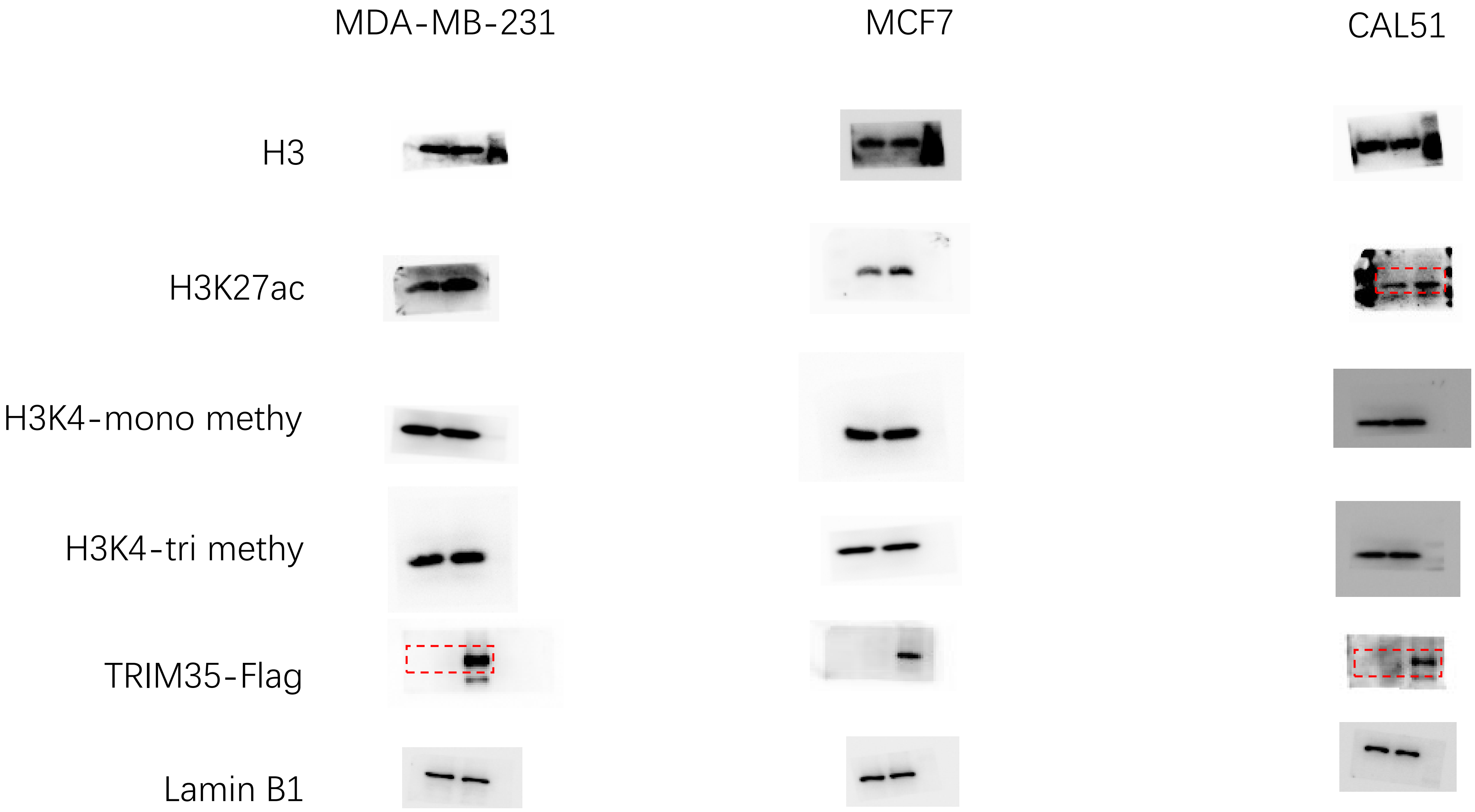
MDA-MB-231
MCF7
CAL51
H3
H3K27ac
H3K4-mono methy
H3K4-tri methy
TRIM35-Flag
Lamin B1

Fig. 4F

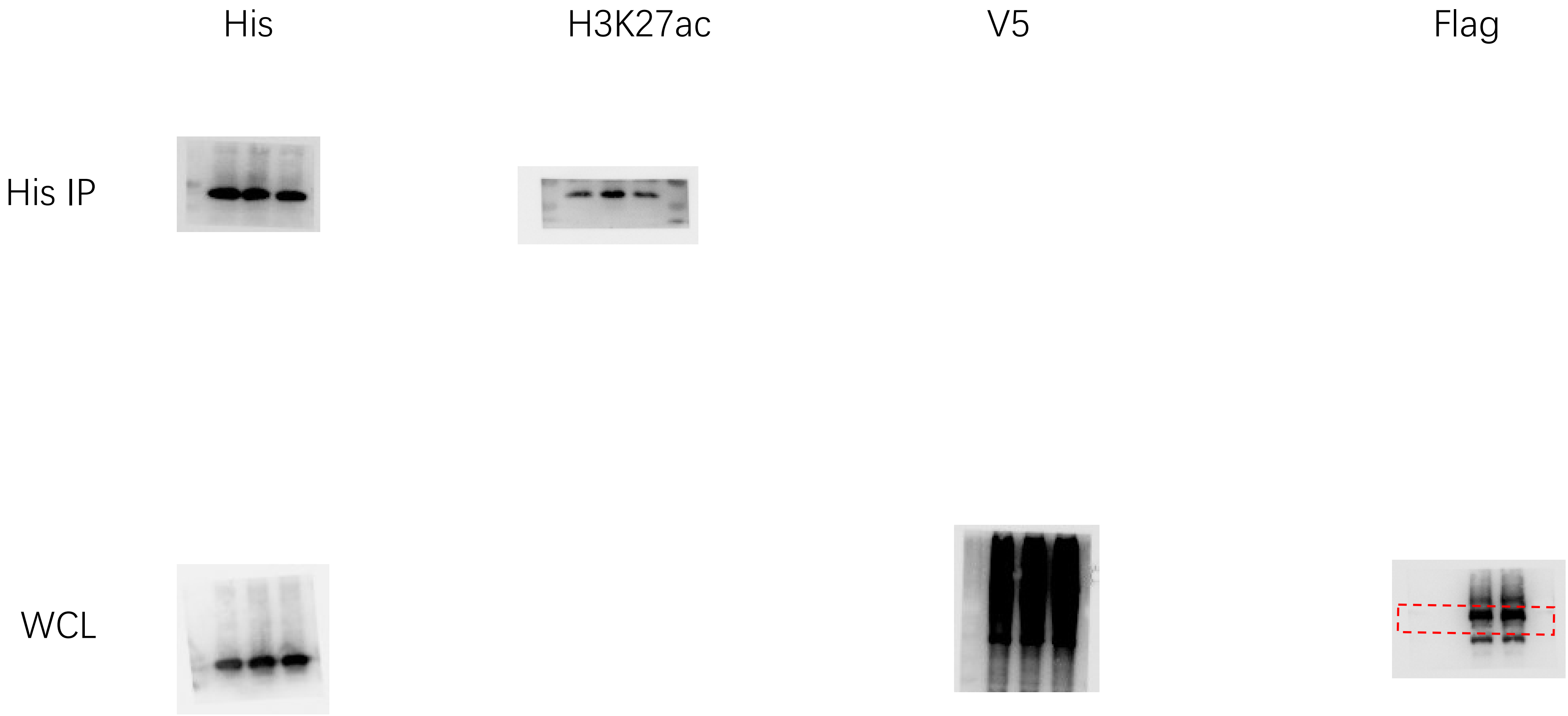

Fig. 4G

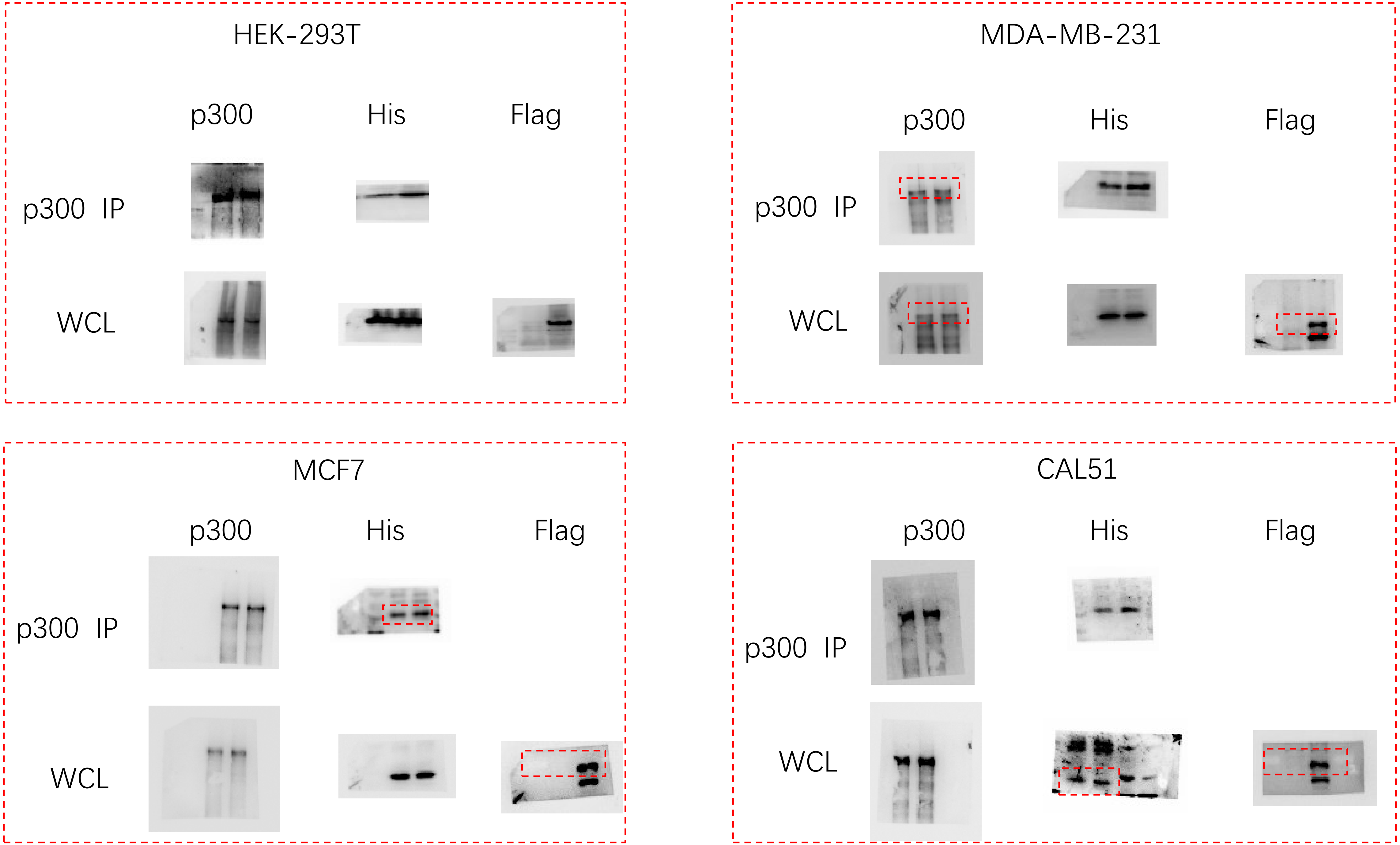
HEK-293T
p300
His
Flag
p300 IP
WCL
MDA-MB-231
p300
His
Flag
p300 IP
WCL
MCF7
p300
His
Flag
p300 IP
WCL
CAL51
p300
His
Flag
p300 IP
WCL

Fig. 4H

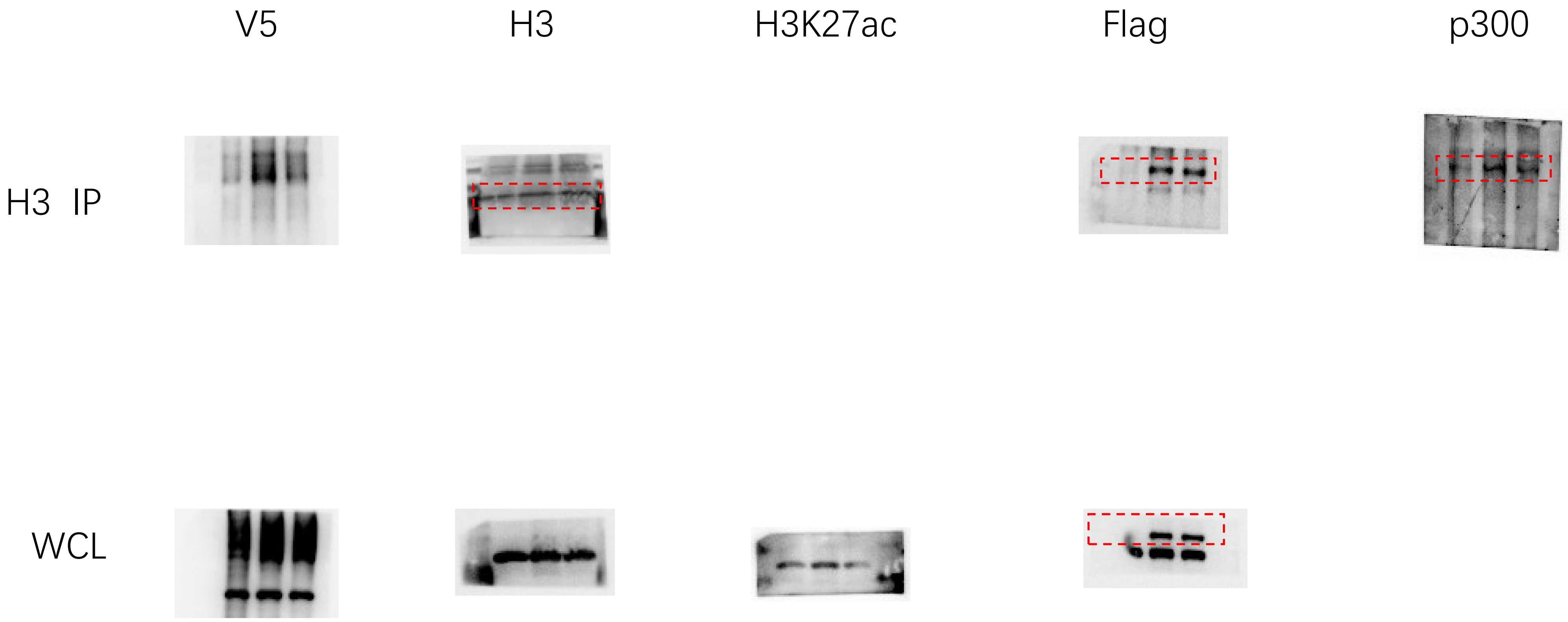

Fig. S2B

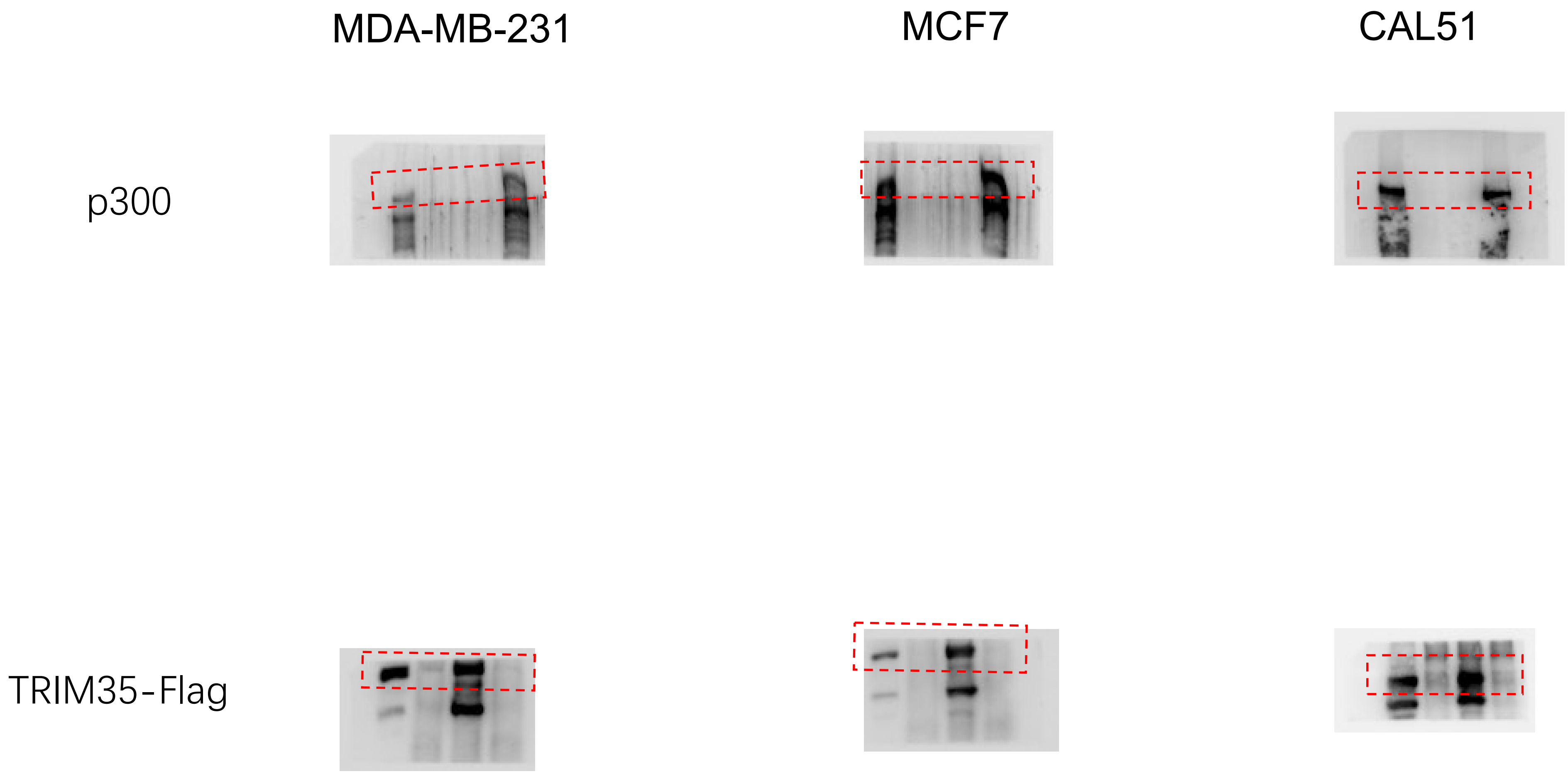

Fig. 6H

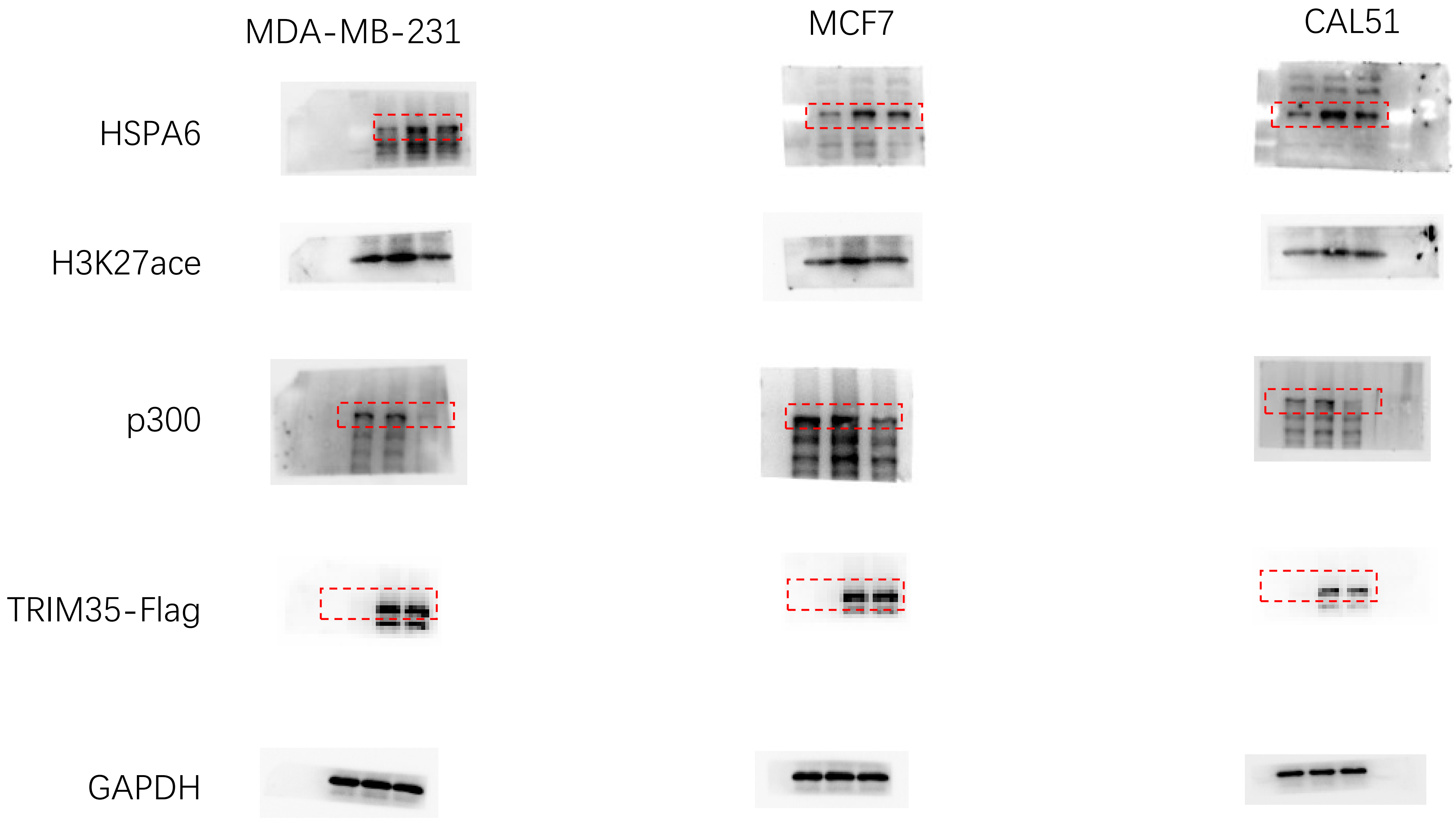

Fig. 7D

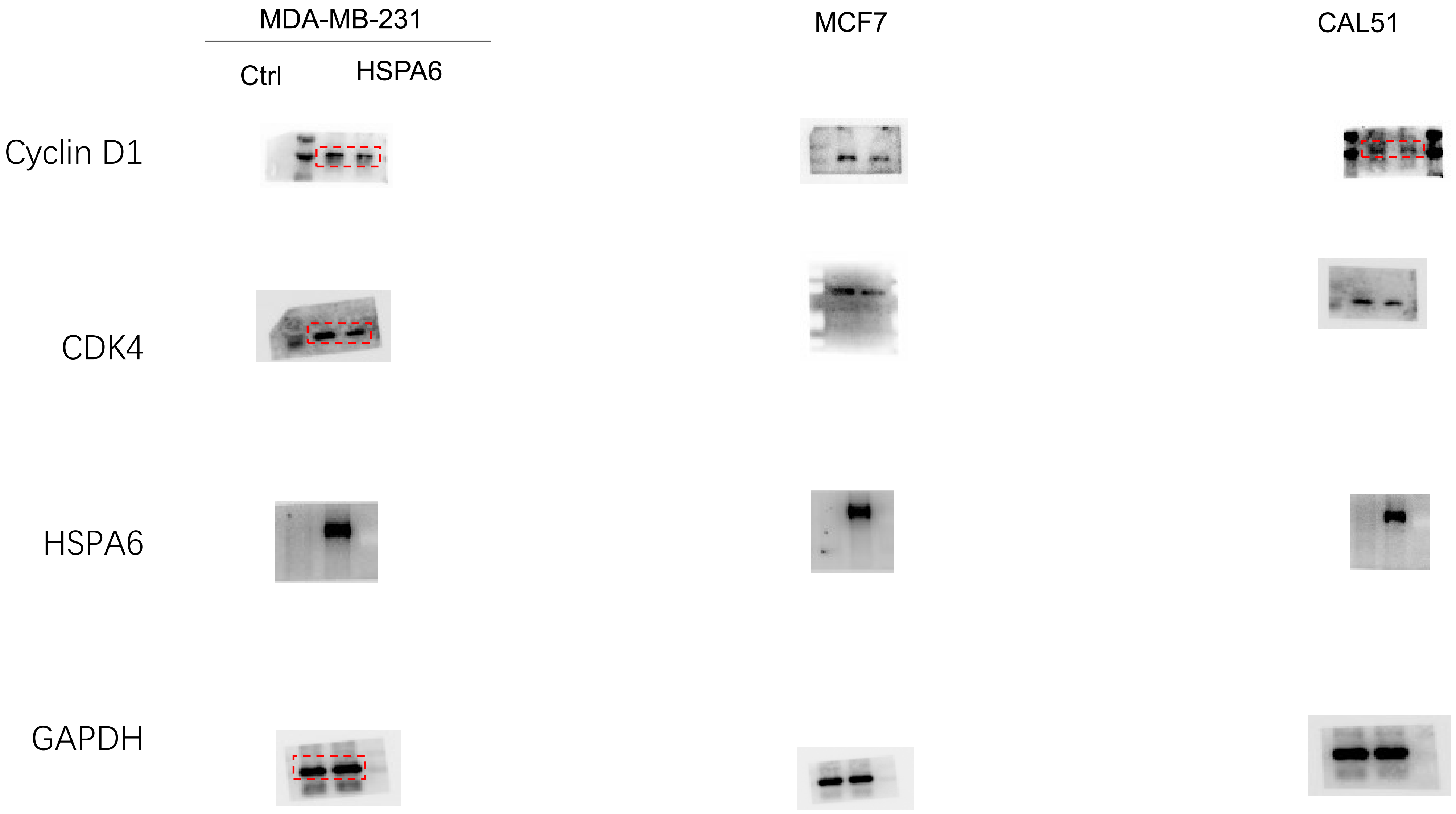

Fig. 7E

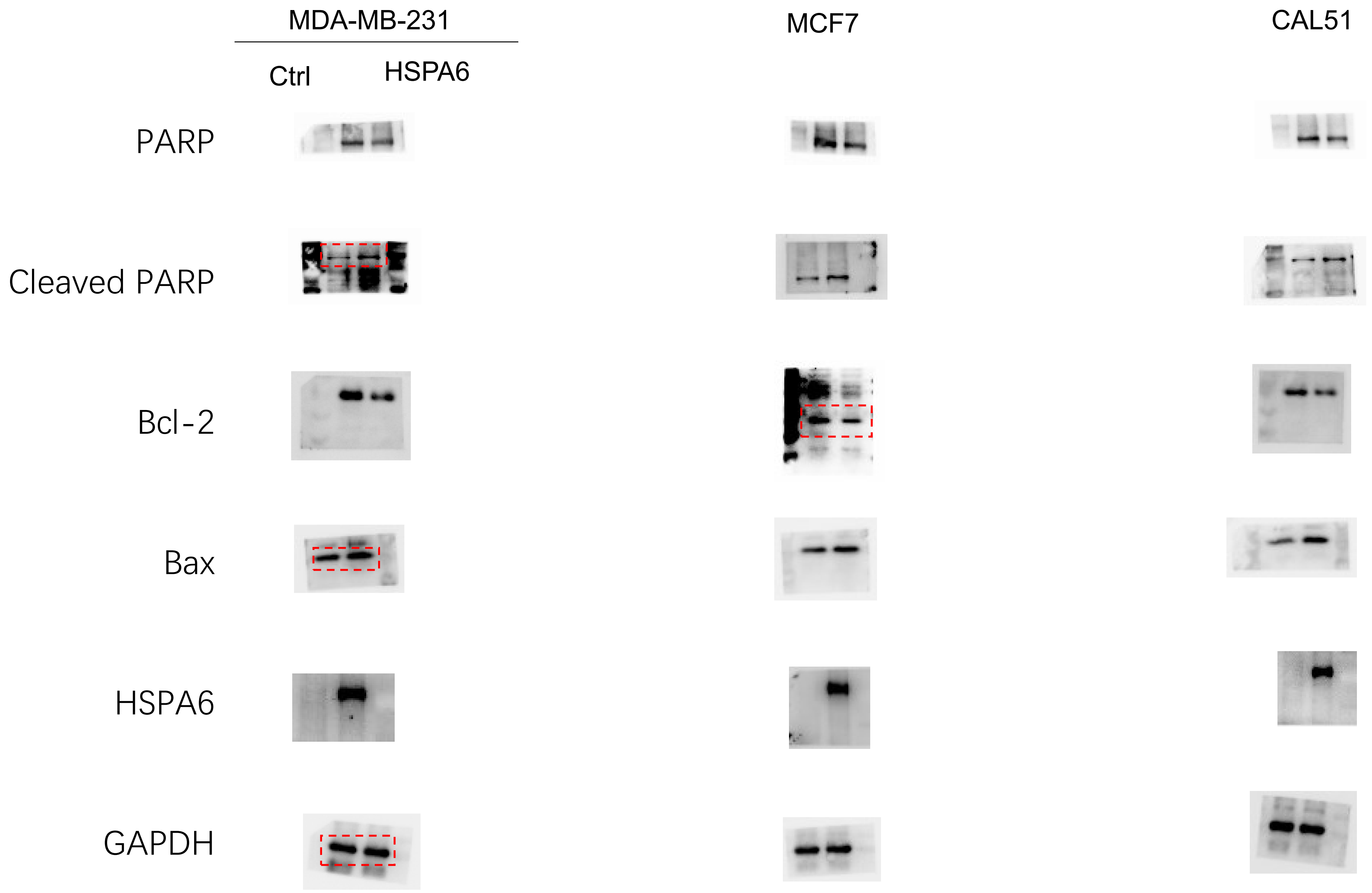
MDA-MB-231
MCF7
CAL51
Ctrl
HSPA6
PARP
Cleaved PARP
Bcl-2
Bax
HSPA6
GAPDH

Fig. S3D

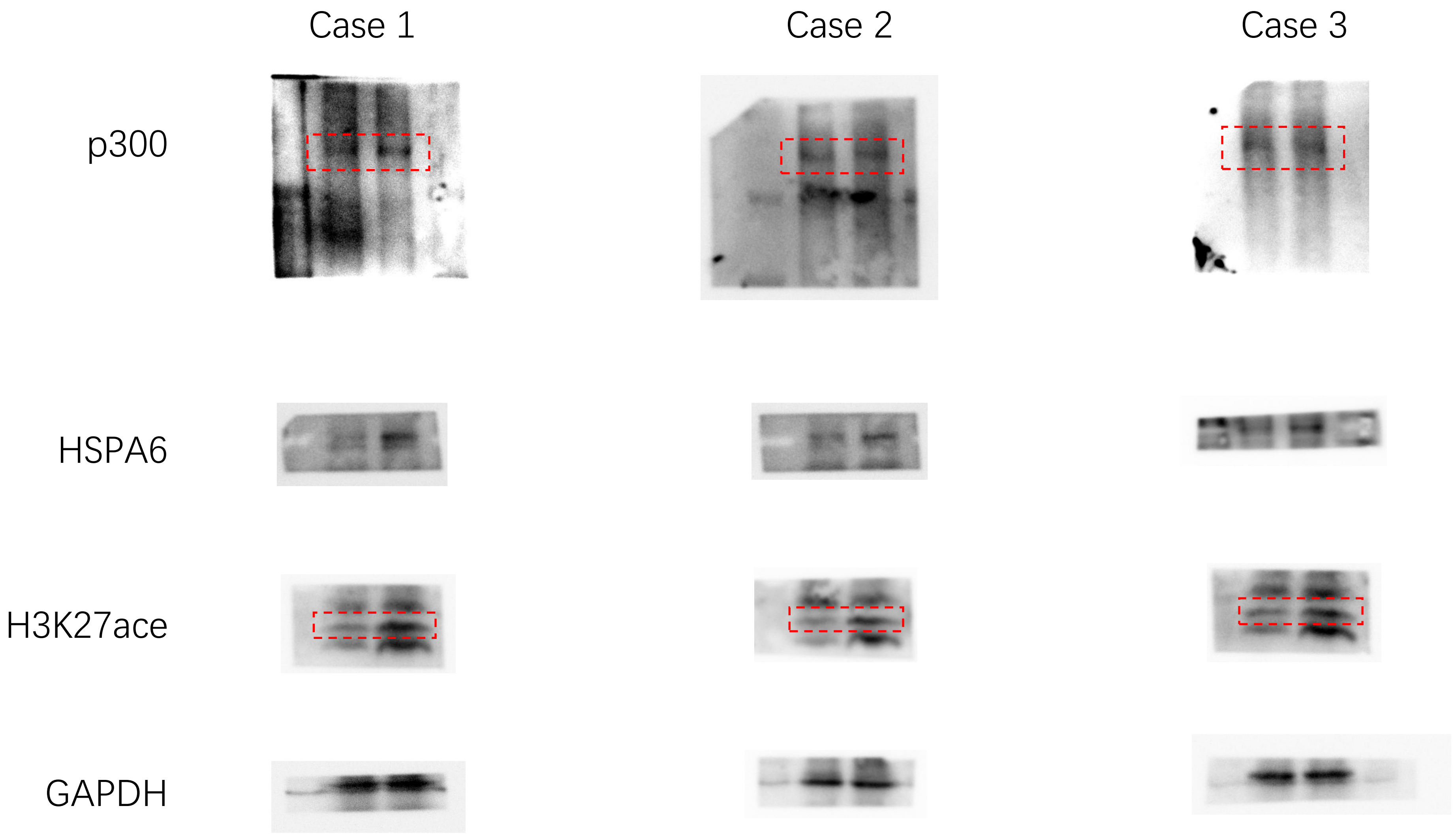

Fig. S4B

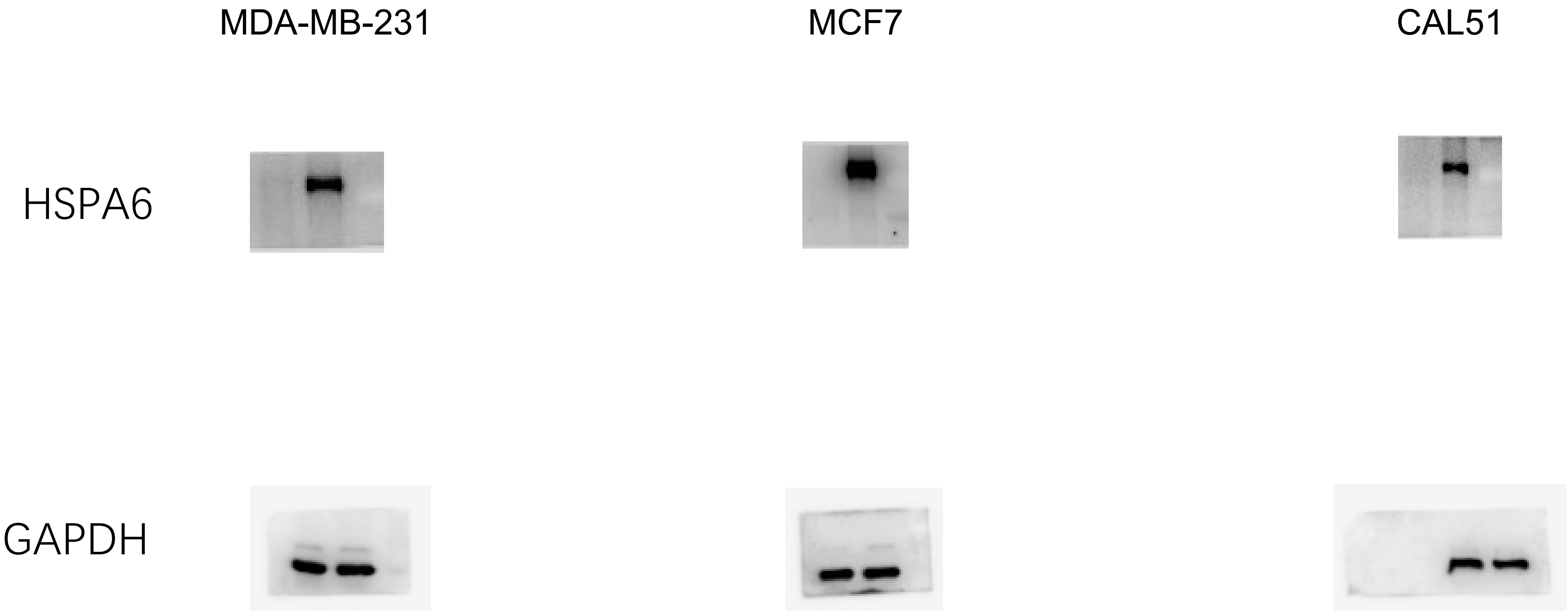

Fig. S4G

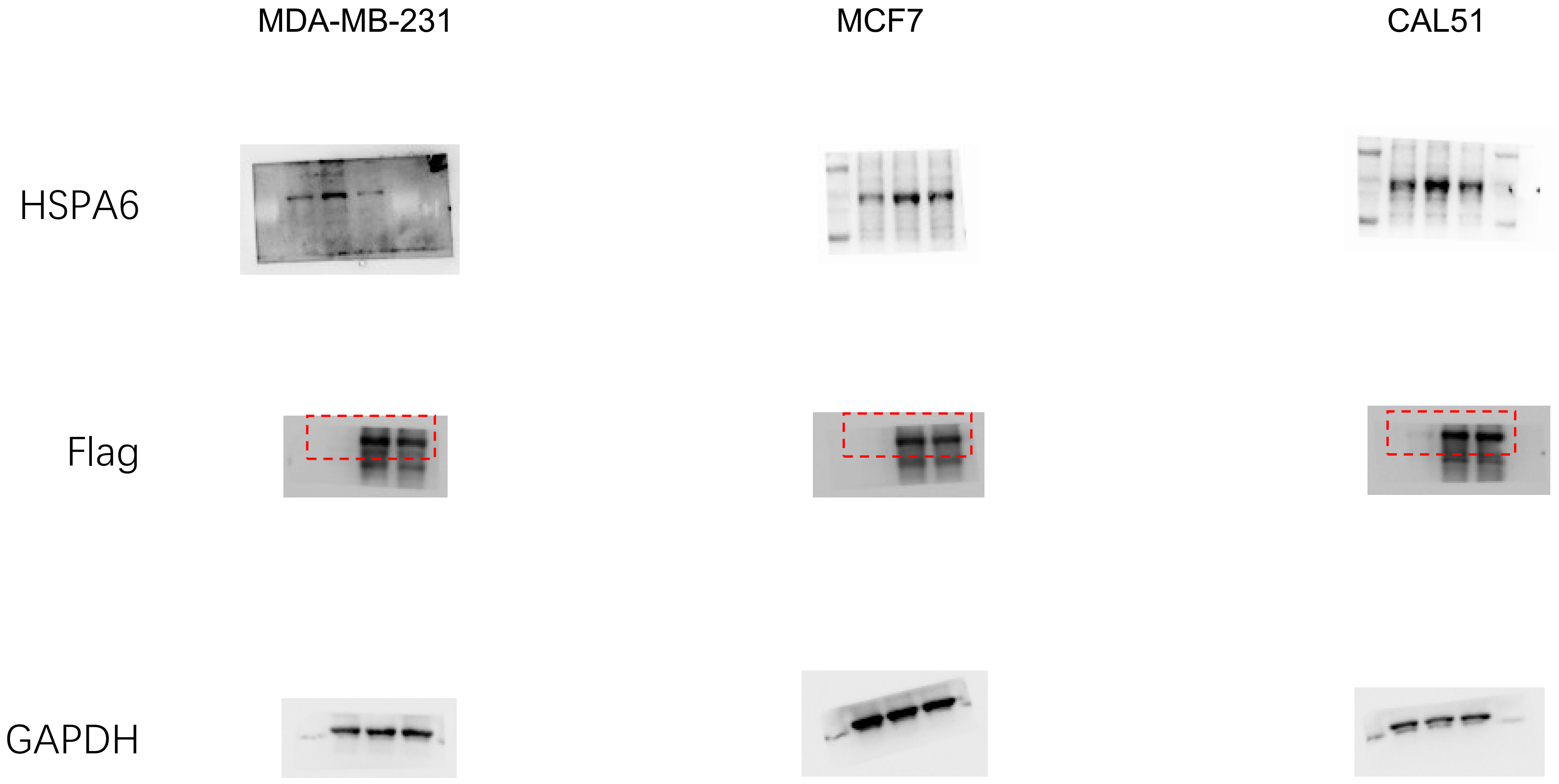

Supplement: Supplementary file 3 — Original western blots [file 41420_2025_2770_MOESM3_ESM.pdf]
